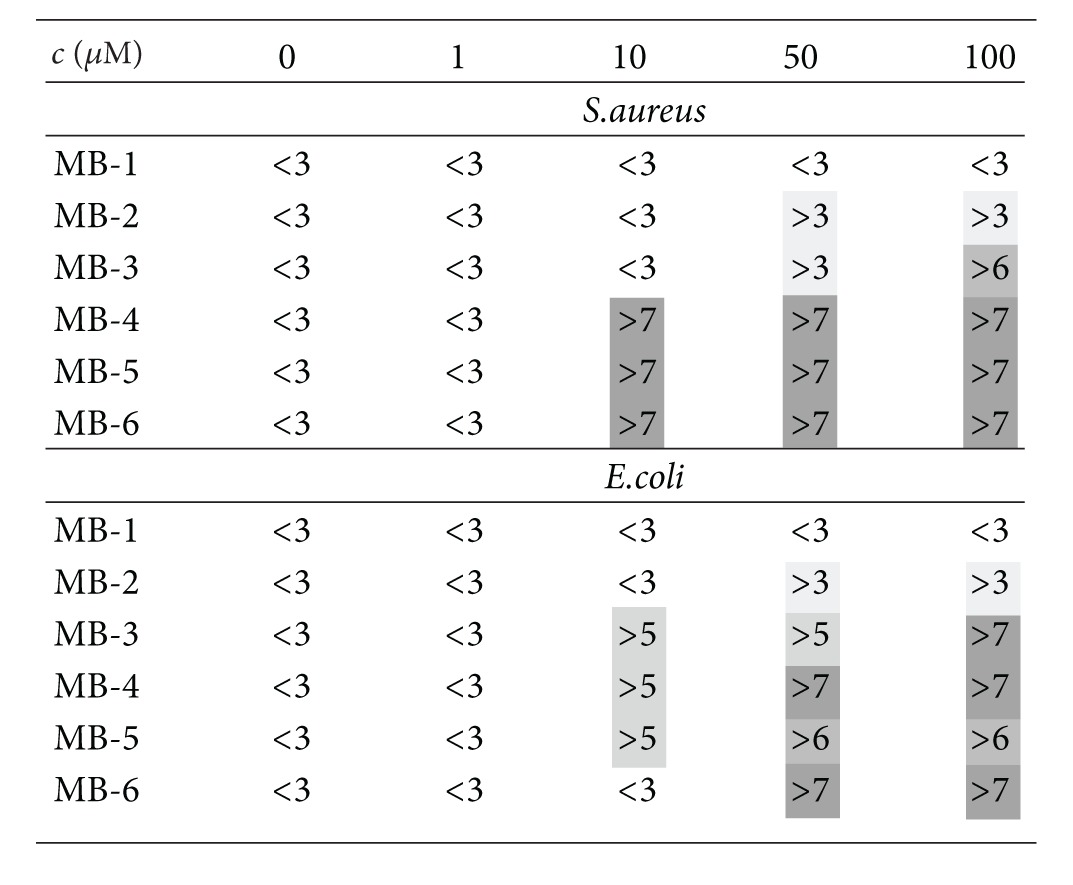# Hydrogen Bond Acceptors and Additional Cationic Charges in Methylene Blue Derivatives: Photophysics and Antimicrobial Efficiency

**DOI:** 10.1155/2013/482167

**Published:** 2012-12-30

**Authors:** Ariane Felgenträger, Tim Maisch, Daniel Dobler, Andreas Späth

**Affiliations:** ^1^Department of Dermatology, University Hospital Regensburg, Franz-Josef-Strauß-Allee 11, 93053 Regensburg, Germany; ^2^Department of Organic Chemistry, University of Regensburg, Universitätsstraße 31, 93053 Regensburg, Germany

## Abstract

Photodynamic inactivation of bacteria (PIB) by efficient singlet oxygen photosensitizers might be a beneficial alternative to antibiotics in the struggle against multiresistant bacteria. Phenothiazinium dyes belong to the most prominent classes of such sensitizers due to their intense absorption in the red-light region (*λ*_abs, max_ ca. 600–680 nm, *ε* > 50000 L mol^−1^ cm^−1^), their low toxicity, and their attachment/penetration abilities. Except simple substituents like alkyl or hydroxyalkyl residues, nearly no modifications of the phenothiaziniums have been pursued at the auxochromic sites. By this, the properties of methylene blue derivatives and their fields of application are limited; it remains unclear if their potential antimicrobial efficacy may be enhanced, also to compete with porphyrins. We prepared a set of six mainly novel methylene blue derivatives with the ability of additional hydrogen bonding and/or additional cationic charges to study the substituents' effect on their activity/toxicity profiles and photophysical properties. Direct detection of singlet oxygen was performed at 1270 nm and the singlet oxygen quantum yields were determined. In suspensions with both, Gram-positive and Gram-negative bacteria, some derivatives were highly active upon illumination to inactivate *S. aureus* and *E. coli* up to 7 log_10_ steps (99.99999%) without inherent toxicities in the nonirradiated state.

## 1. Introduction

Owing to its structure, methylene blue (MB, **7**) can penetrate cells and can be used as a staining agent in histology [1, 2] or as a chemotherapeutic [3–6]. Binding with cytoplasmic structures within the cell and interference with oxidation/reduction processes [7, 8] may lead to killing of bacteria, funguses, viruses, or parasites.

Methylene blue and its derivatives were proven to be versatile photosensitizers for the inactivation of pathogens in suspension [9–11], for example, *Candida* species [12, 13], *E. coli* [14, 15], *S. aureus* [16] and *MRSA* [17], tropical diseases [18], or several viruses [19, 20], and therefore finds its application in antimicrobial fields, like blood disinfection [21–24]. *In vivo* the phenothiazinium chromophore system is used with benefit against oral infections [25] performing photodynamic root channel disinfection [26–29] or treatment of periodontitis, inactivating bacteria like *E. faecalis* [30], *P. gingivalis* [31], or *A. actinomycetemcomitans* [32]. As “state of the art” it may be given that methylene blue and its derivatives like 1,9-dimethylmethylene blue (DMMB), toluidine blue (TBO) and new methylene blue (NMB) can achieve a log reduction >6 log steps of a bacterium at fluences ranging from 0 to 30 J cm^−2^, using a fluence rate of 125 mW cm^−2^ in a concentration range of 2 to 10 *μ*M in suspension [33].

Although methylene blue and its alkyl- and aryl-derivatives are well studied [34–37], not many approaches followed variations of the structure beyond simple modifications [38]. For example, the effect of additional positive charges on the antimicrobial activity and the influence of such substituents on the singlet oxygen yield have not been investigated yet. A comparison of such photosensitizers with related hydrogen bond acceptor moieties, thus also strongly increasing polarity, is lacking as well as the direct spectroscopic determination of the group's influence on the singlet oxygen quantum yield. More polarity in the structure should cause the molecules to stay outside cell, causing only photodynamic damage of the cell wall. Positive charges in addition may lead to better attachment to the exterior of the cell, resulting in shorter process times and higher antimicrobial activity in comparison to methylene blue. Attack from outside the cell also overcomes the problem of reduced photosensitizer activity by efflux mechanisms [39].

The synthesis of methylene blue and its derivatives was summarized [41] and the preparation of phenothiazinium systems with additional positive charges for other purposes was demonstrated [42], but a straight forward, reliable purification protocol without the use of expensive HPLC methods is still missing.

**Figure 1:**
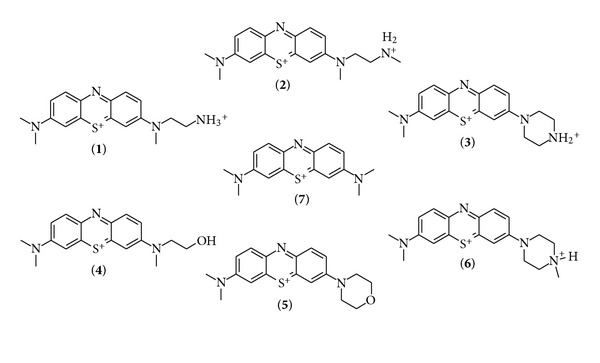
Compounds investigated (**1–6**) in comparison to the lead compound methylene blue (MB, **7**); counterions are chloride in all cases and were avoided for clarity [40].

We focused on the synthesis and study of methylene blue derivatives with highly polar and/or hydrophilic groups, to extend the field of highly hydrophilic phenothiazinium compounds in antimicrobial photodynamic therapy (aPDT). One substituent in the methylene blue lead structure (**7**) was changed (Figure 1) in order to achieve pursuable variations of the behaviour of the compound. 

## 2. Material and Methodology

### 2.1. Synthesis, Purification, and Analytics

Methylene blue has been purchased by Sigma Aldrich and was purified by flash chromatography with silica gel using dichloromethane/ethanol 10 : 1 as the eluent mixture resulting in an overall pureness of >99% (HPLC-MS). Methylene blue and its derivatives were dissolved and diluted in H_2_O and kept in the dark at 4°C until use. Further information on chemicals, analytics, description of the syntheses and purification protocols are given in the supporting information. See the supporting information available online at http://dx.doi.org/10.1155/2013/482167.

### 2.2. Absorption Spectroscopy

Absorption spectra were recorded at room temperature with a DU640 spectrophotometer (Beckman Instruments GmbH, Munich, Germany) in a concentration range of 5·10^−6 ^M to 1·10^−4 ^M. The transmission has been measured and the absorption cross-section *σ* [cm²] was calculated according to the following equation:

(1) $$\begin{array}{c} \sigma =-\frac{\ln\left(T/100\right)}{c\cdot l\cdot N_{A}} \end{array}$$

with *σ* being the absorption cross section, *c* the concentration of PS, *l* the length of light path through the solution, *T* the transmission in %, and *N*_*A*_ the Avogadro constant.

### 2.3. Direct Detection of Singlet Oxygen Luminescence

Solutions with the photosensitizer were filled in a quartz cuvette with a path length of 1 cm (QS-101, Hellma Optik, Jena, Germany) and were excited during magnetic stirring with an OPO tuneable laser (EKSPLA, Lithuania) at a wavelength of *λ* = 600 nm, power output *P* = 90 mW, frequency of *f* = 1 kHz, and therefore an energy per pulse of *E* = 9 · 10^−5^ J. Every sample was illuminated with 20.000 pulses. Direct detection as described in previous papers was done by time resolved measurements at 1270 nm (10 nm FWHM filter) in near-backward direction with respect to the exciting beam using an infrared-sensitive photomultiplier (R5509-42, Hamamatsu Photonics Deutschland GmbH, Herrsching, Germany). The luminescence intensity is given by

(2) $$\begin{array}{c} I\left(t\right)=\frac{c}{t_{R}^{-1}-t_{D}^{-1}}\left[\exp\left(-\frac{t}{t_{D}}\right)-\exp\left(-\frac{t}{t_{R}}\right)\right], \end{array}$$

where *c* was used to fit the singlet oxygen luminescence signal, and *t*_*R*_ and *t*_*D*_ are the rise and decay times [44–46]. Therefore, the Levenberg-Marquardt algorithm of Mathematica (Wolfram Research, Champaign, IL USA) was used. The luminescence signal was spectrally resolved using interference filters in front of the photomultiplier tube at wavelengths ranging from 1150 nm to 1400 nm. The values show the integrated luminescence signals detected at a certain wavelength and are normalized to the maximal value. A Lorentz-shaped curve was fitted through the measurement points, with the maximum at *λ* = 1275 nm [47].

### 2.4. Quantum Yield of Singlet Oxygen Formation

The quantum yields (Φ_Δ_) of the derivatives of MB were compared to the Φ_Δ_ of MB which is reported in the literature being Φ_Δ_ = 0.52 in aqueous solution [48]. Therefore, a sample of each photosensitizer was diluted to a final absorption of *A* = 30% at *λ* = 600 nm in H_2_O. 3 mL (O_2_ concentration at air-saturation at 25°C) of each sample was illuminated in a quartz cuvette (path length of 1 cm) with the OPO tuneable laser with the above given parameters, and the emitted singlet oxygen photons were determined by the integral over the luminescence curve.

### 2.5. Photostability

The photosensitizers were diluted to a final absorption of *A* = 30% at the wavelength of *λ* = 600 nm. The samples were irradiated in quartz cuvettes at a path length of 1 cm with the OPO tuneable laser at the given parameters with 180000 laser pulses during magnetic stirring. After the irradiation absorption spectroscopy was done in the range from 200 nm to 1000 nm and the data was compared to the nonilluminated samples.

### 2.6. Bacterial Strains

The biochemical analysis of each bacteria strain was done by a VITEK2 System (bioMérieux, Nürtingen, Germany) according to NCCLS (National Committee for Clinical Laboratory Standards) guidelines. The bacterial strains, *S. aureus* (ATCC 25923) and *E. coli* (ATCC 25922), were grown aerobically at 37°C in Mueller-Hinton broth (Gibco Life Technologies GmbH, Eggenstein, Germany). A 500 *μ*L portion of an overnight cell culture (5 mL) was transferred to 50 mL of fresh BHI media and grown at 37°C on an orbital shaker. When the cultures reached the stationary phase of growth, the cells were harvested by centrifugation (200 g, 15 min), washed with phosphate-buffered saline (PBS; Biochrom, Berlin, Germany) at pH 7.4, containing 2.7 mM KCl and 0.14 M NaCl, and suspended in PBS at an optical density of 0.6 at 600 nm corresponding to *≈*10^8^ – 10^9^ cells/mL for the use in the phototoxicity experiments. 

### 2.7. Light Source

The bacteria were illuminated using an incoherent light source PDT1200 provided by Waldmann Medizintechnik (Villingen-Schwenningen, Germany) which covers partially the absorption spectrum of methylene blue and its derivatives (Figure 3). The normalized emission spectrum of the light source was provided by Waldmann Medizintechnik. The maximal fluence rate at the level of the illuminated samples was 50 mW cm^−2^. The samples were illuminated for 10 min (30 J cm^−2^). In order to estimate the effectiveness of the uptake of the light energy by the different derivatives the values of the emission spectrum “*Em*” were folded with the values for the absolute absorption “*Abs*” for the spectral region between 500 and 800 nm. According to the following formula an effective toxicity “Eff.Tox.” was predicted for each derivative:

(3) $$\begin{array}{c} \text{Eff.Tox.}=\;\left(\sum_{i=500\;\text{nm}}^{800\;\text{nm}}{\text{Em}}_{i}\cdot {\text{Abs}}_{i}\right)\cdot \Phi_{\Delta }. \end{array}$$

Here it has been taken into account that the effectively absorbed energy (i.e., the sum of the product of emission and absorption) of every photosensitizer is used partially to generate singlet oxygen. Therefore, also the quantum yield Φ_Δ_ was multiplied to the effectively absorbed energy. The results, given as percentaged values, are listed in Table 1.

### 2.8. Phototoxicity Assay of the Bacteria

A bacterial cell number of 10^8^ to 10^9^ mL^−1^ was incubated for 10 min in the dark with different concentrations of methylene blue-based photosensitizers (0, 1 *μ*M, 10 *μ*M, 50 *μ*M, and 100 *μ*M). At the end of the incubation period the cells were transferred into a 96-well microtitre plate (200 *μ*L/well) and illuminated for 10 min (50 mW cm^−2^; 30 J cm^−2^). Controls were neither sensitized with a photosensitizer nor exposed to the light source or were incubated with the photosensitizer only. After illumination, the survival of the bacteria was determined by CFU assay. Serially diluted aliquots of treated and untreated (no photosensitizer, no light) cells were plated on Mueller-Hinton agar and the numbers of CFU mL^−1^ were counted after 24 h of incubation at 37°C. 

### 2.9. Data Analysis and Statistics for Cell Experiments

Each individual experiment was performed at least in triplicate. All primary data are presented as means with standard deviation of the mean. A reduction of at least 3 orders of magnitude of log_10_ viable median numbers of bacteria cells was considered biologically relevant with regard to the guidelines for hand hygiene [49].

## 3. Results

In order to cover the field of hydrophilic phenothiaziniums in our studies, we selected a small library of methylene blue derivatives to investigate the influence of hydrogen bond acceptors (**4** and **5**) and/or an additional cationic charge (**1**, **2**, **3,** and **6**) located in one of the systems side chains on the photophysical characteristics and the antimicrobial efficiency. This selection allows us to study the effect of the substituent's structure on the stability of the methylene blue derivative comparing cyclic (compounds **3**, **5**, and **6**) with acyclic (compounds **1**, **2**, and **4**) moieties. The effect of the nature of the additional charge, being either a tertiary (**6**) or a secondary (**2** or **3**) or a primary (**1**) ammonium group, can be compared in this selection. All photosensitizers were supplied in their chloride form to ensure comparability of the photosensitizer salts and avoiding disadvantageous influence of the counterion on the phototoxicity studies. As trifluoroacetate is known to be toxic against microorganisms, it has to be exchanged with a nontoxic counterion. Iodide salts readily react with singlet oxygen to form triiodide, which has a negative influence on the antimicrobial efficacy of the photosensitizer [50]. Figure 1 summarizes the studied compounds.

### 3.1. Synthesis

**Scheme 1:**
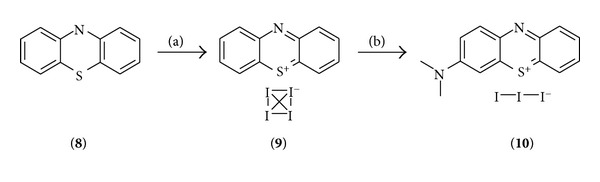
Synthesis of the precursors; conditions: (a) DCM, I_2_, RT, 2 h, quant.; (b) HNMe_2_, MeOH, RT, 14 h, 63%.

3-Dimethylamino-phenothiazinium triiodide (**10**) was proven to be a suitable starting material for synthesis of the chromophore library. It was prepared using known conditions from literature starting from phenothiazine (**8**), as can be seen in Scheme 1 [40].

**Scheme 2:**
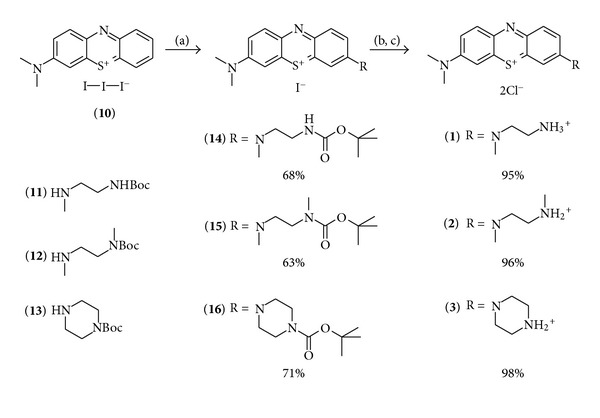
Synthesis of the boc-protected derivatives (**14**–**16**) and their transformation to the deprotected chromophores as chloride salts (**1**–**3**); conditions: (a) DCM, boc-protected amine (**11**, **12,** or **13**), NEt_3_, RT, 5 h; (b) DCM, TFA, RT, 4 h; (c) ion exchanger Amberlite IRA958, water.

The compound was converted to the desired products (**14**, **15**, and **16**) in good yields using an excess of the appropriate boc-protected amine (**11**, **12**, or **13**) in presence of triethylamine in dichloromethane. After deprotection with TFA using standard conditions, the counterion was exchanged versus chloride using amberlite IRA958. Both steps resulted in quantitative yields (Scheme 2).

**Scheme 3:**
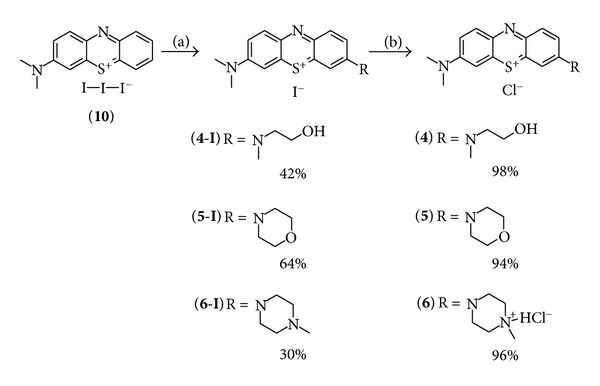
Synthesis of the second set of methylene blue derivatives as chloride salts (**4**–**6**); conditions: (a) DCM, secondary amine: 2-(N-methylamino)ethanol, morpholin or 4-N-methyl-piperazine, RT, 5 h; (b) ion exchanger Amberlite IRA958, water, then HCl.

The second set of phenothiazinium compounds was prepared using similar conditions and reacting 2-(N-methylamino)ethanol to give **4-I**, morpholin to yield **5-I** or 4-N-methyl-piperazine to give **6-I**, respectively, with moderate to good yields. After purification by flash chromatography and crystallisation, the counterion was exchanged with chloride in quantitative yield following the same protocol as before (Scheme 3). 

For detailed synthesis and purification protocols, see the supporting information. 

### 3.2. Photophysical Data

#### 3.2.1. Absorption Spectra for Different Photosensitizer Concentration

Phototoxic reactions of methylene blue on microorganisms can involve redox reactions between the dye and the pathogen, or the generation of reactive oxygen species (ROS) via type-I mechanism or type-II mechanism, for example, direct energy transfer from excited triplet state of the photosensitizer to oxygen, resulting in the formation of singlet molecular oxygen [21, 51]. Both mechanisms are described to be important for an antibacterial effect. The photoinactivation of bacteria might therefore be dependent on the aggregation state of the molecule (dimerisation) [52–55] that can be influenced also by the presence of bacteria [56, 57] or other influences like the pH value of the surrounding [13, 58, 59]. Dimerization of methylene blue and some of its derivatives like toluidine blue (TBO) has been described [60, 61] and also has an influence on the photophysical properties of the dye resulting in different phototoxic efficacies [56]. This has been investigated for the new methylene blue derivatives in H_2_O within a concentration range from 10 to 200 *μ*M. 

**Figure 2:**
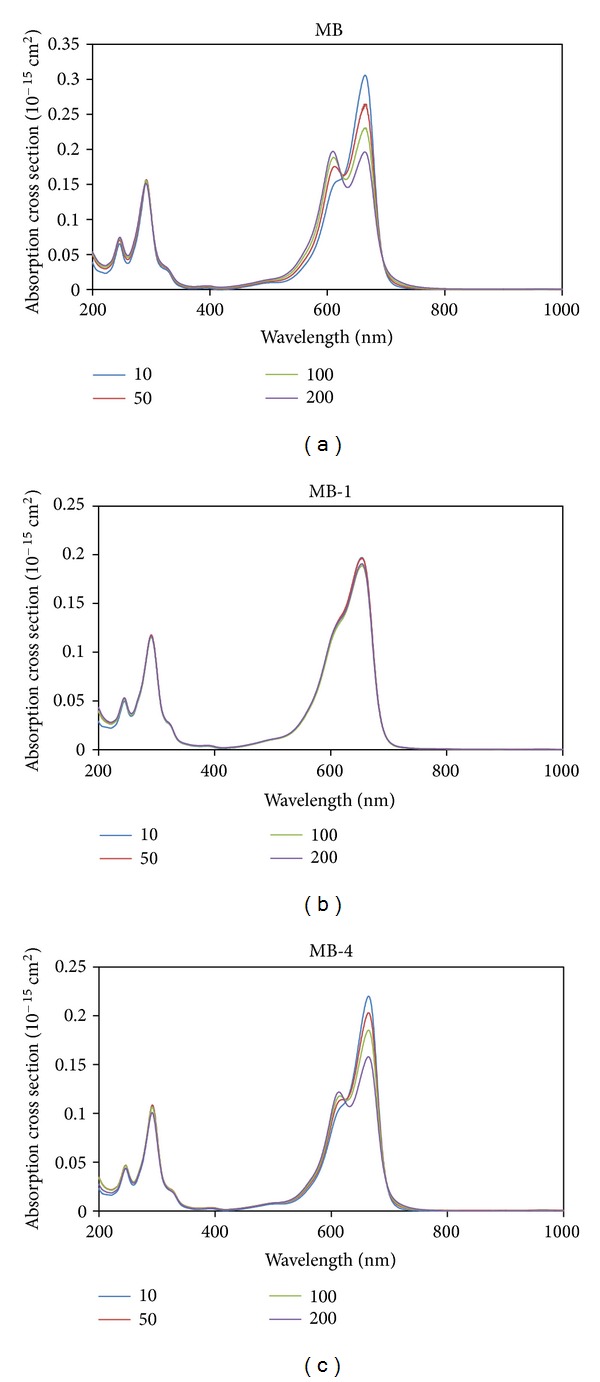
Absorption spectra of MB and its derivatives for different concentrations. Absorption spectra of methylene blue and its derivatives MB-1 and MB-4 within a concentration range of 10–200 *μ*M in H_2_O; the measurements show dimerisation for MB, MB-4, and MB-5 in the given concentration range (see supporting information).

In Table 1 the absorption maximum of each derivative is shown. MB-4 and MB-5 closely match the peak of methylene blue. In the given concentration range the derivatives MB-4 and MB-5 show the formation of an absorption peak at 613 nm (Figure 2). The peak between 662 and 664 nm is diminished with increasing the dye concentration (hypochromicity). The evolving local maximums at 613 nm show each a hypsochromic effect indicating aggregation processes. The peak at 613 nm is considered to be the dimer, as described for methylene blue [60, 61].

#### 3.2.2. Absorption of the Lamp Emission by the Different Derivatives of MB

**Figure 3:**
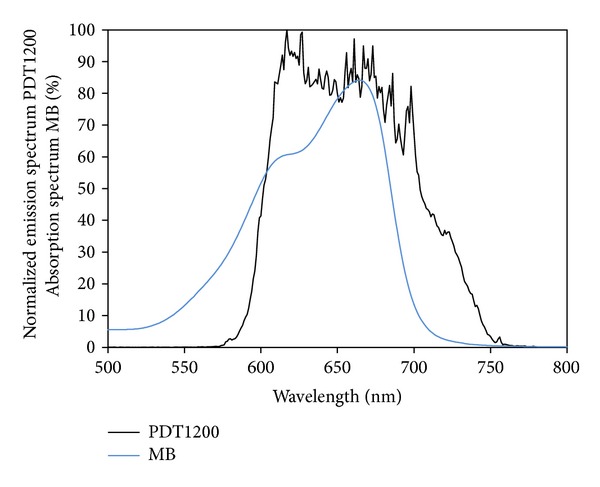
Emission spectrum of the PDT1200. Normalized (100%) emission spectrum of the PDT1200 and absorption spectrum of methylene blue at a concentration of 10 *μ*M in H_2_O showing the percentaged absorption. An overlap of these two spectra was calculated by folding the values for emission and absorption for each wavelength and summing up these values.

The emission spectrum of the incoherent light source PDT1200 covers partially the absorption spectrum of methylene blue and its derivatives (Figure 3). The effectiveness of light absorption at the same molar concentration of 10 *μ*M was calculated with (3) and the results are listed in Table 1. There one finds the “*overlap*” of emission and absorption and the effective toxicity *“*Eff.Tox.,*”* which estimates the phototoxic effect on microorganisms via singlet oxygen by taking into account the relative singlet oxygen quantum yield Φ_Δ_^1.00^, which describes the part of absorbed energy that generates singlet oxygen. “Eff.Tox.” describes therefore the predicted effective toxicity that was calculated by multiplication of Φ_Δ_^1.00^ with the value of the overlap. With this method we assume methylene blue being most active and in descending manner MB (54%) > MB-4 (44%) > MB-1 (37%) > MB-5 (36%) > MB-3 (35%) > MB-2 (28%) > MB-6 (20%).

#### 3.2.3. Photostability

**Figure 4:**
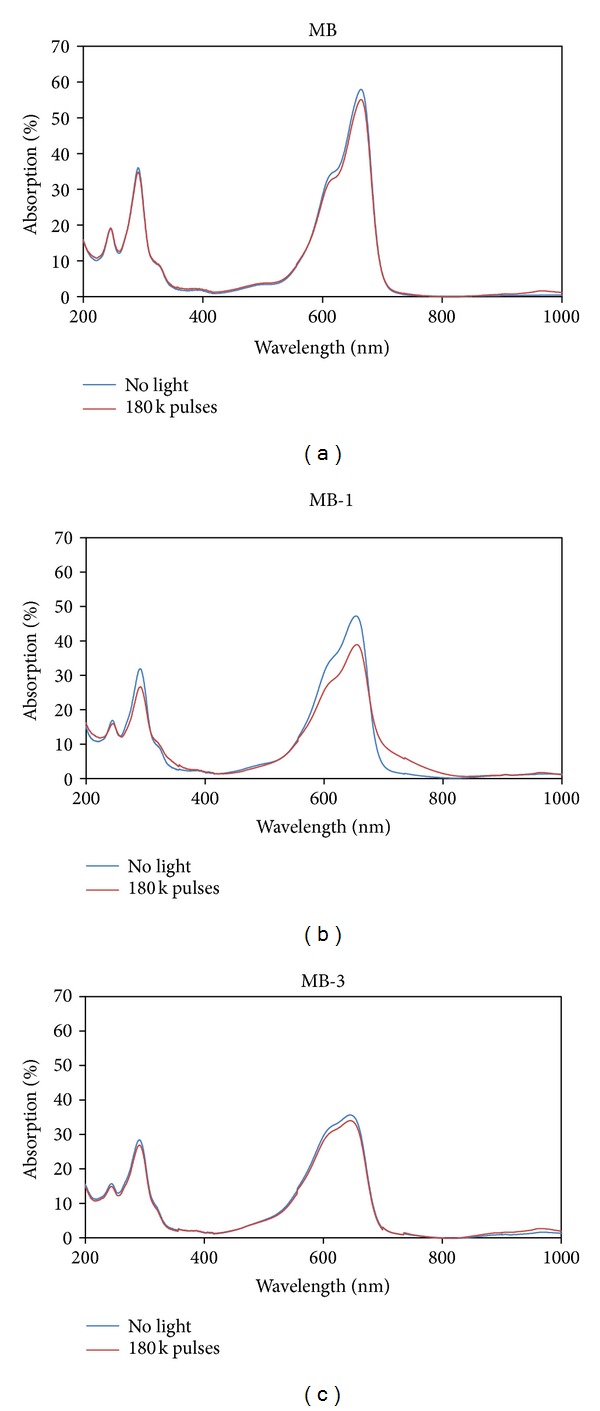
Photostability of MB and its derivatives. Photostability measurements in a quartz cuvette with an irradiation at 600 nm with 180000 laser pulses; exemplarily methylene blue and its derivatives MB-1 and MB-3 are shown; only MB-1 and MB-2 show a decrease in the absorption in the visible and in the UV range (see supporting information).

With diluting methylene blue and its derivatives to a final absorption of *A* = 30% at 600 nm the same amount of light energy per time unit is absorbed by each derivative. After irradiation at 600 nm with 180000 laser pulses (=3 min), resulting in an energy of *E* = 16.2 J, the derivatives MB-1 and MB-2 showed a decrease in their main absorption region and in the UV-range, while MB and the other derivatives showed photostability (Figure 4, Table 1, see supporting information). The value to estimate photostability was given with the ratio of the absorption maxima after irradiation and before irradiation. The photophysical measurements such as time- and spectrally resolved singlet luminescence did not exceed the amount of energy used for the photostability testing.

#### 3.2.4. Time and Spectrally Resolved Singlet Oxygen Luminescence

**Figure 5:**
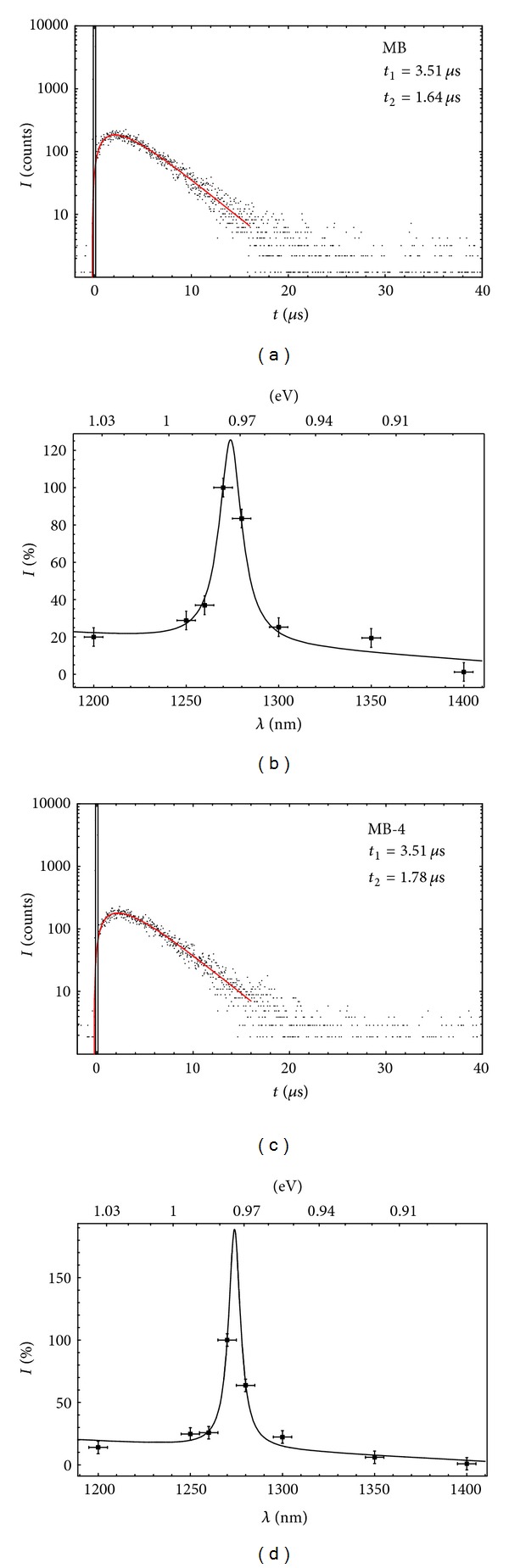
Time- and spectrally resolved singlet oxygen luminescence. Time- and spectrally resolved singlet oxygen luminescence of methylene blue and exemplarily its derivative MB-4 in air saturated H_2_O at 25°C; singlet oxygen is generated and detected at 1275 nm with a decay time of *≈*3.5 *μ*s with all derivatives (see supporting information).

Singlet oxygen luminescence was generated by all derivatives of methylene blue and was detected time and spectrally resolved in an air-saturated solution of H_2_O at 25°C. 20 k laser pulses equals an irradiation time of 20 s. Each time-resolved luminescence signal showed a rise and decay time, whereas the rise time differed for each derivative but the decay time was around 3.5 *μ*s, confirming the values in literature for the decay of singlet oxygen in aqueous surrounding [43]. The rise and decay times, *t*_*R*_ and *t*_*D*_, are shown in Table 1. The maximum of the singlet oxygen phosphorescence was detected at 1275 ± 5 nm (Figure 5).

#### 3.2.5. Quantum Yield of Singlet Oxygen Formation

The quantum yields for singlet oxygen formation of the derivatives of methylene blue have been compared in air saturated H_2_O to the quantum yield of methylene blue, since it has been described that the quantum yield can be higher in basic environment [58]. Each photosensitizer absorbed the same amount of energy within the same irradiation time. Furthermore, the same amount of oxygen in the water surrounding of the molecule was given in order to deactivate the excited triplet state of the photosensitizer. Therefore, the singlet oxygen photons give evidence of the effectiveness of each derivative. In Table 1 the results for the estimation of the quantum yields Φ_Δ_ of singlet oxygen for all derivatives are summarized relative to the literature value of the quantum yield for methylene blue of 0.52 [43]. The quantum yield of MB was then set to 1.00 in this paper, as described by Φ_Δ_^1.00^, because it is only needed for reference and comparison purposes. For the values of the quantum yield an error of 10% in regard to the measurement procedure was assumed. Taking into account that the photostability of MB-1 and MB-2 is not given within a range of 180000 laser pulses we consider an irradiation with 20000 laser pulses for the quantum yield measurement as an insignificant change in the absorption spectrum (*≈*2%). The Φ_Δ_ of MB-4 is comparable to the yield of methylene blue, whereas the other yields are smaller (Table 1, see supporting information). 

### 3.3. Photobiological Activity

**Figure 6:**
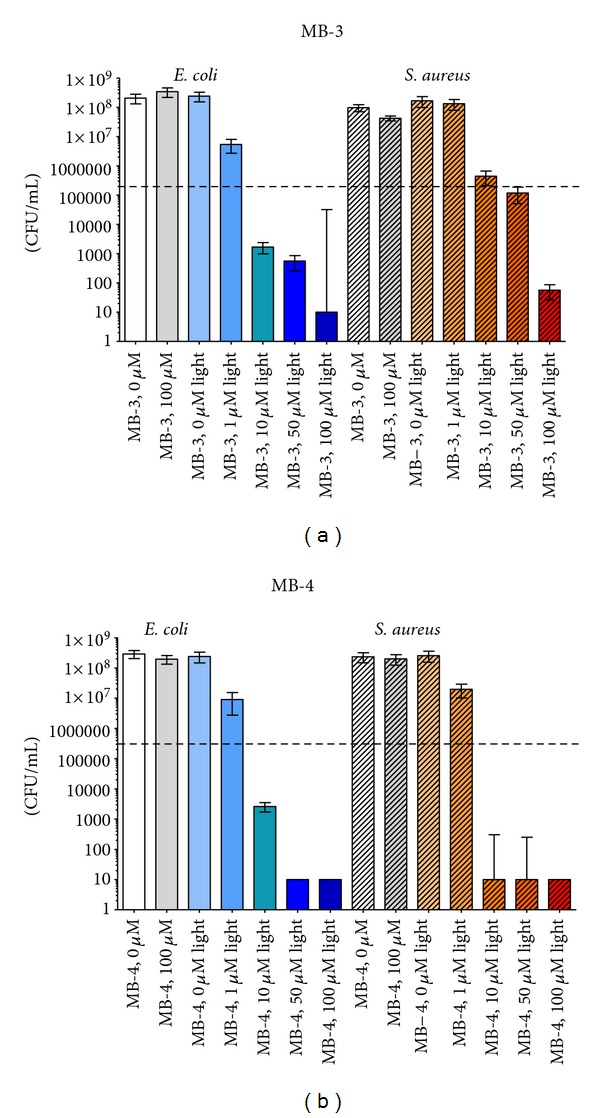
Photodynamic inactivation of *E. coli* and *S. aureus* by MB-3 and MB-4. Photodynamic treatment was performed using different concentrations of MB-3 or MB-4 with and without illumination (30 J cm^−2^). Surviving colonies were counted 24 h later. Black-dashed line corresponds to a reduction of 3 log_10_ steps in bacteria numbers (99.9% killing efficacy). White and grey column: controls without illumination. Colored columns: MB-3 + light activation, blue: *E. coli*; crosshatched: *S. aureus*. (*n* = 3 experiments: mean values ± standard deviation).

The irradiation of the Gram-positive *S. aureus* and the Gram-negative *E. coli* upon incubation with different concentrations (0–100 *μ*M) of MB-1, MB-2, MB-3, MB-4, MB-5, and MB-6 caused a decrease in viability of CFU/mL (Figure 6 and supplementary figure) except for MB-1. Light activation of MB-1 achieved only a reduction of viable bacteria numbers of both bacteria strains of *≈*1 log_10_ (supplementary figure). Furthermore, MB-2 induced only an antibacterial activity of 99.9% using a concentration of 50 *μ*M upon light activation. MB-3 showed a better killing efficacy as compared to MB-2 upon light activation. However, light-activated MB-3 achieved a killing efficacy of >99.9% at a concentration of 50 *μ*M against both strains, whereas MB-4, MB-5, and MB-6 exhibit the greatest killing rate of >99,999% (5 log_10_ steps) after irradiation with a concentration >10 *μ*M (Figure 6 and supplementary figure). All bacterial samples that were incubated without photosensitizers exhibited normal growth with or without irradiation, demonstrating that the maximal fluence rate (50 mW cm^−2^) at the level of the irradiated samples alone had no antibacterial effects. An overview of the killing rates after irradiation can be found in Table 2. In summary, MB-4, MB-5, and MB-6 killed more efficiently both *S. aureus* and *E. coli* compared to MB-1 and MB-2.

## 4. Discussion

The synthetic protocol presented allows the preparation of methylene blue derivatives with high yields (up to 70%), matching the highest values found in literature [42]. 

The photobiological activity of methylene blue for *S. aureus* and *E. coli* was described in terms of minimal lethal concentration for 10^6^ cells/mL by Wainwright et al. [10, 62]. To achieve this killing efficacy a concentration of 1 *μ*M for *S. aureus* and 100 *μ*M for *E. coli* at an applied energy dose of 6.3 J cm^−2^ was necessary. Therefore, the phototoxicity data of the derivatives of MB presented in this report are comparable to the toxicity of MB as described in literature. 

The photobiological activities of the derivatives of methylene blue show some dependencies on their photophysical behavior and their chemical properties. The structure-response principle, for example, the influence of the substituents on the phototoxicity, can be derived from the following considerations. We expected lowered dimerization ability in compounds carrying the additional cationic charge (**1**, **2**, **3**, and **6**) due to higher charge repulsion, a higher antimicrobial efficacy of these compounds due to better attachment to the cell wall, and an increased stability of cyclic derivatives in comparison to open chain analogs. The last two points were expected to be also true for both compounds carrying the hydrogen bond accepting oxygen substituent (**4** and **5**).

The quantum yield of the photosensitizers gives a value for its efficacy to generate singlet oxygen. But it takes not into account the effective uptake of the light energy. Therefore, the prediction of effective toxicity considering the absorbed light energy by the photosensitizers in combination with their quantum yield is a more realistic value to describe a possible biological killing efficacy. Nevertheless for this study it turned out only for some derivatives to be in line with the measured photobiological activity. Compounds **4** and **1** had a high ranking, predicting effectiveness, whereas (**6**) had a low “Eff.Tox.” value (Table 1). The toxicity data for (**4**) are in line with the calculations but (**1**) and (**6**) show each the opposite behaviour; while (**1**) did not have any toxic effect at all, but was expected to show toxicity upon irradiation comparable to (**4**), (**6**) was expected to show a low phototoxicity, but inhibited the microorganisms very effectively (up to 7 log_10_ steps = 99.99999%). 

The antimicrobial efficacy of the derivatives that equipped with additional charge in the side chain rises gradually, starting from the open chain substituted compounds **1** and **2**, going to the compounds with cyclic substituent **3** and **6** (for *S. aureus*: **1** < **2** < **3** < **6** and for *E.coli* **1** < **2** < **3** ~ **6**, Table 2, see also supporting information), and is in good accordance with the calculated “Eff.Tox.” value, except compound **6** (Table 1). Since **1** and** 2** show a decrease in their absorption spectrum when being illuminated (see Table 1), the absorbed amount of light energy decreases as well, which might result in a lower generation of singlet oxygen compared to the photostable derivatives (stability: **1** = **2** ≪ **3** = **6**) (Table 1, see also supporting information). Therefore, a lower phototoxicity compared to the stable derivatives might be the result.

The data show, moving from primary to secondary to tertiary ammonium charges, that an efficiency increase can be achieved. This may be due to better polarity characteristics facilitating attachment or even uptake. Also, the ease of deprotonation of the groups in aqueous media is assumed to have influence. The pKs values of tertiary ammonium groups (**6**) are lower than that of secondary (**2** and **3**), which in turn are again lower than that of the primary groups (**1**). The charges can be seen as more permanent in a pH equilibrium in solution moving from primary to tertiary groups and therefore might have more influence on the attachment to the bacterial cell wall, which is in agreement with the data of the phototoxicity experiments (**6** > **3** > **2**) again. Better cell attachment governed by the additional, more permanent charge might be the main reason for the higher efficacy of compound **6** in comparison to all others, although this was not expected from the calculated value for “Eff.Tox.” (Table 1). Part of the antimicrobial effect can origin from redox chemistry damaging the bacterial cell wall and can be a reason for the higher efficacy of this compound, despite it is only showing a low value for singlet oxygen generation (Table 1).

Free amine bases can be oxidised in solution by singlet oxygen. The more the protonation/deprotonation equilibrium lies on the side of the free base, the more accessible the compound might be for degradation upon illumination. This can be a reason for the lower photostability of compounds **1** and **2** (Table 1, suppoting information).

As expected, compounds **1**, **2**, **3**, and **6** show a lowered ability to dimerize, (Table 1, Figure 2). This can be seen as benefit of the additional positive charge in this class of compounds. It enables the use of these phenothiazinium photosensitizers in a broader concentration range in comparison to methylene blue.

Compounds **4** and **5** are comparable in their stability and ability to dimerize, also matching the values of these parameters of methylene blue (**7**). Both photosensitizers with hydrogen bond accepting moieties show a high activity against *S. aureus* and *E. coli* in the photodynamic inactivation studies (up to 7 log_10_ steps = 99.99999%, Figure 6, Table 2), whereas the antimicrobial efficacy of **4** is slightly higher than that of **5** against *E. coli* and comparable for *S. aureus*. This is in good agreement in regard to their singlet oxygen quantum yield and the predicted effective toxicity.

In its activity compound **6** can be compared to **4 **and **5**; all reach up to 7 log_10_ steps bacteria inactivation (99.99999%) already below the 50 *μ*molar concentration range. A better linking to the cell wall or even uptake into bacteria is therefore supposed for these derivatives. Therefore, it is interesting to follow the uptake mechanism of each derivative and to investigate the phototoxicity after washing procedures following the incubation process with the photosensitizers in a future study.

## 5. Conclusion

In this study new derivatives of methylene blue derived from modifications of the substituents of methylene blue were described and investigated on their effectiveness for aPDT. One substituent in the methylene blue lead structure (**7**) was changed, in order to achieve pursuable variations of the compound. We focused on the synthesis and study of derivatives with highly polar and/or hydrophilic groups and prepared the compounds in high purity as chloride salts. For this purpose we successfully revised the literature known syntheses and supplied straight forward protocols for preparation and purification of the photosensitizers.

A structure-response relationship was described from a chemical point of view, based on spectroscopic measurements and on investigations of the photobiological activity against *S. aureus* and *E. coli*. Our results point towards a positive influence on the antimicrobial efficacy by hydrogen acceptor bond moieties and additional tertiary charges in the substituent of methylene blue derivatives achieving 7 log_10_ steps for *S. aureus* and *E. coli* at concentrations of 10 *μ*M with 10 min of irradiation. This can compete with the best examples of known antimicrobial photodynamic agents like porphyrins. The singlet oxygen quantum yields of some compounds are comparable to the yield of methylene blue without overtopping it. Dimerisation of such photosensitizers in solution in a broad concentration range can be suppressed by introducing additional positive charges in the side chains.

A simple method of estimating the effective phototoxicity by taking singlet oxygen quantum yield in combination with the absorbed light energy into account was presented for the new derivatives. Not for all derivatives this value for the effective phototoxicity is in line with the data of the killing rates. Some exhibit a high killing rate which is not supported by spectroscopic data and *vice versa*. Therefore, other mechanisms of action have to be assumed and the adhesion to bacteria cell walls and the uptake of the derivatives has to be investigated in a further study, including washing experiments following the incubation period.

## Supplementary Material

A set of mainly novel methylene blue derivatives was prepared, supplying a straight forward and reliable synthesis and purification protocol. The effect of their ability of additional hydrogen bonding and/or the effect of additional cationic charges on their activity/toxicity profiles and photophysical properties was investigated. Some derivatives were higly active upon illumination to inactivate *S. aureus* and *E. coli* up to 7 log_10_ steps. No inherent toxicities were observed in the non-irradiated state.

## Figures and Tables

**Table 1:** Characteristic values of methylene blue and its derivatives MB-1 to MB-6, where *λ*_max_ describes the maximum of the absorption; the dimerisation was detected in a concentration range between 10–200 *μ*M; the photostability is described with the ratio of the height of the absorption maximum after irradiation to height of the maximum before irradiation with 180000 laser pulses; *t*_*R*_ and *t*_*D*_ are the rise and decay time of the time resolved singlet oxygen luminescence, respectively; Φ_Δ_ is the quantum yield of singlet oxygen generation relatively to the quantum yield of methylene blue, which is found in literature to be 0.52 [43]; Φ_Δ_^1.00^ is the quantum yield of MB set to 1.00, to simplify the comparison. For the values of the quantum yield an error of 10% in regard to the measurement procedure had to be estimated. “overlap” describes the uptake of the lamp emission spectrum by the different photosensitizers at a concentration of 10 *μ*M. “Eff.Tox.” describes therefore the predicted effective toxicity that was calculated by multiplication of Φ_Δ_^1.00^ (ref. MB) with the value of the overlap.

PS	*λ*_abs,max_	dimeriz.	photostab.	*t*_*D*_ (*μ*s)	*t*_*R*_ (*μ*s)	Φ_Δ_^1.00^ (ref. MB)	Φ_Δ_^1.00^ (ref. MB)	Overlap [%]	Eff.Tox. [%]
MB	664	Yes	95%	3.51	1.64	0.52*	1.00	54.4	54
MB-1	653.5	No	82%	3.47	1.93	0.45 ± 0.05	0.87	43.1	37
MB-2	650.5	No	81%	3.57	1.76	0.38 ± 0.04	0.73	38.7	28
MB-3	643.5	No	95%	3.44	1.92	0.47 ± 0.05	0.90	38.4	35
MB-4	663.5	Yes	97%	3.51	1.78	0.51 ± 0.05	0.98	44.5	44
MB-5	662	Yes	96%	3.46	1.70	0.41 ± 0.04	0.79	45.4	36
MB-6	649	No	95%	3.47	1.76	0.35 ± 0.04	0.67	29.6	20

**Table 2:** Overview of the phototoxic efficacy of the MB derivatives on *S.  aureus* and *E. coli*; the table shows only the photodynamic treatment with light (effects of dark toxicity can be found in the supporting information). Different concentrations of each photosensitizer were applied and toxic efficacy is described in steps of log_10_-reduction; therefore “<3” means a reduction <3 log_10_ steps (<99.9%).