# Supplementary material for: Hydrogen Bond Acceptors and Additional Cationic Charges in Methylene Blue Derivatives: Photophysics and Antimicrobial Efficiency
Source: Biomed Res Int. 2012 Dec 30;2013:482167. doi: 10.1155/2013/482167 (PMC3591237; doi:10.1155/2013/482167)
Supplement: Supplementary file 1 — A set of mainly novel methylene blue derivatives was prepared, supplying a straight forward and reliable synthesis and purification protocol. The effect of their ability of additional hydrogen bonding and/or the effect of additional cationic charges on their activity/toxicity profiles and photophysical properties was investigated. Some derivatives were higly active upon illumination to inactivate S. aureus and E. coli up to 7 log10 steps. No inherent toxicities were observed in the non-irradiated state. [file 482167.f1.pdf]

Hydrogen bond acceptors and additional cationic charges in methylene blue derivatives – photophysics and antimicrobial efficiency

Ariane Felgenträger[1], Tim Maisch[1], Daniel Dobler[2] and Andreas Späth[2,3]*

SUPPORTING INFORMATION

Synthesis an Purification of the compounds

Materials and methods

Analytical characterization of the synthesized compounds was done by common methods. Melting Points were determined on Büchi SMP or a Lambda PhotometricsOptiMelt MPA 100 and are uncorrected. IR Spectra were recorded with a Bio-Rad FT-IR Excalibur FTS 3000 equipped with a Specac *Golden Gate* Diamond Single Reflection ATR-System. Absorption spectra were recorded on a Varian Cary BIO 50 UV/VIS/NIR spectrometer with temperature control using 1 cm quartz cuvettes (Hellma) and Uvasol solvents (Merck, Baker or Acros). Fluorescence measurements were performed with UV-grade solvents (Baker or Merck) in 1 cm quartz cuvettes (Hellma) and recorded on a Varian 'Cary Eclipse' fluorescence spectrophotometer with temperature control. Electro spray mass spectra were performed on a Finnigan MAT TSQ 7000 ESI-spectrometer. Other Mass Spectra were recorded on Varian CH-5 (EI), Finnigan MAT 95 (CI; FAB and FD); Xenon serves as the ionization gas for FAB. NMR spectra were recorded on BrukerAvance 600 (^{1}H: 600.1 MHz, ^{13}C: 150.1 MHz, T = 300 K), BrukerAvance 400 (^{1}H: 400.1 MHz, ^{13}C: 100.6 MHz, T = 300 K) or BrukerAvance 300 (^{1}H: 300.1 MHz, ^{13}C: 75.5 MHz, T = 300 K) relative to external standards. NMR spectra were recorded in $CDCl_3$ at 300 MHz (^{1}H) or 75 MHz (^{13}C) unless stated otherwise. Characterization of the signals: s = singlet, d = doublet, t = triplet, q = quartet, m = multiplet, bs = broad singlet, dd = double doublet, dt = double triplet, ddd = double double doublet.

[1] Department of Dermatology, University of Regensburg, Franz Josef Strauss Allee 11, Regensburg 93042, Germany

[2]Department of Organic Chemistry, University of Regensburg, Universitätsstrasse 31, Regensburg 93053

[3]Corresponding author: Fax: Int +49-941-944-8943

E-mail address: andreas.spaeth@chemie.uni-regensburg.de

Integration is determined as the relative number of atoms, the coupling constants are given in Hertz [Hz]. The multiplicity of the carbon atoms is given as (+) = CH_3 or CH, (-) = CH_2 and (C_{quat}) for quaternary carbon atoms. Error of reported values: chemical shift: 0.01 ppm for ^{1}H-NMR, 0.1 ppm for ^{13}C-NMR and 0.1 Hz for coupling constants. The solvent used is reported for each spectrum. Analytical TLC plates (silica gel 60 F_{254}) and silica gel 60 (70-230 or 230-400 mesh) were used for chromatographic separations. Visualization of the spots was by UV light and/or staining with ninhydrin in ethanol. PE means petrol ether with a boiling range of 70 - 90° C. All other solvents and chemicals were of reagent grade and used without further purification. 1-(tert.-Butoxycarbonyl)piperazine was purchased from TCI Europe in a purity of > 98% and phenothiazine was purchased from Aldrich in a purity of > 98%. Both were used as received.

Synthesis and Purification

The mono-boc-protected diamines **11** and **12** were prepared by nucleophilic substitution of an appropriate alkylbromide (**E1**) with methylamine or by boc-protection of commercially available 1,2-dimethyl-ethylendiamine (**E2**).

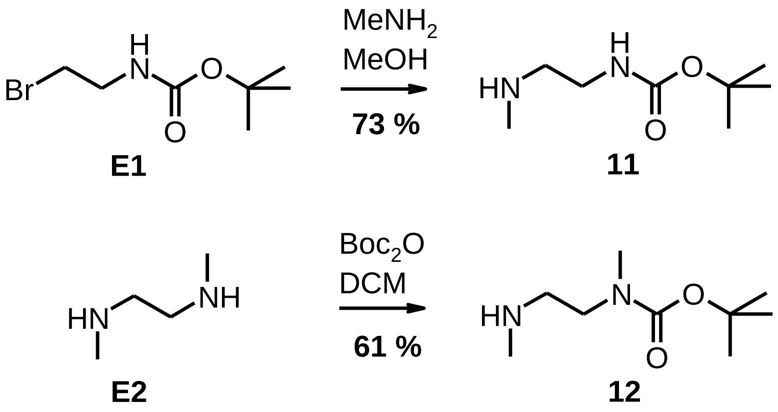

Scheme S-1: Preparation of the boc-protected side chain building blocks

*2-(N-butyloxycarbonyl-2-aminoethyl)-1-(methyl)amine (**11**) (literature known, improved procedure)*[i]

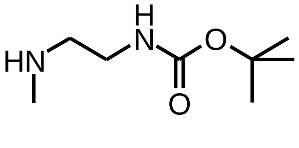

tert-butyl [2-bromoethyl]carbamate[ii] (2.23 g, 10 mmol) in methanol (100 mL) was slowly dropped in a vigorously stirring, ice-cold solution of methylamine in methanol (40 %, 50 mL) over a period of 2h, keeping the temperature between 2-5°C. After stirring over night at room temperature, the solvent and the excess amine were removed at reduced pressure. The crude material was purified by column chromatography with silica gel using chloroform/methanol 10:1 → 6:1 as the eluent, to give the bromide salt of **11** as colourless solid (1.83 g, 7.26 mmol, 73 %).

^{1}H-NMR (300 MHz, MeOD): δ [ppm] = 3.39 (2H, t, *J* = 6.4 Hz), 3.11 (2H, t, *J* = 6.4 Hz), 2.71 (3 H, s), 1.43 (9 H, s); - **MS** (ESI-MS, CH_2Cl_2/MeOH + 10 mmol NH_4OAc): e/z (%) = 175.1 (100, MH^+), 119.1 (56, MH^+ - C_4H_9);

A solution of this salt in dichloromethane (50 mL) was washed four times with diluted aqueous sodium hydroxide solution (4x 20 mL, 2 M). The organic layer was separated and dried over $MgSO_4$. The solvent was removed at reduced pressure to give the free base **11** as colourless oil (1.22 g, 7.06 mmol, 97 %).

^{1}H-NMR (300 MHz, $CDCl_3$): δ [ppm] = 5.43 (1 H, bs, NH), 3.09 (2 H, m), 3.52 (2 H, t, *J* = 6.4 Hz), 2.14 (3 H, s), 1.36 (1 H, s), 1.32 (9 H, s); - **^{13}C-NMR** (75 MHz, $CDCl_3$): δ [ppm] = 155.3 (C_{quat}), 77.9 (C_{quat}), 50.2 (-), 38.9 (-), 35.0 (+), 27.4 (+); - **MS** (ESI-MS, CH_2Cl_2/MeOH + 10 mmol NH_4OAc): e/z (%) = 175.1 (100, MH^+), 119.1 (43, MH^+ - C_4H_9); - **MW** = 174.24 g/mol; **MF** = $C_8H_{18}N_2O_2$

*2-(N-butyloxycarbonyl-methylamino)ethyl-1-methylamine (**12**) (literature known, improved procedure)*[iii]

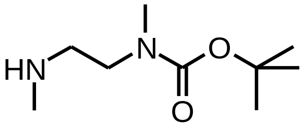

A solution of 1,2-dimethyl-ethylendiamine (8.60 g, 100 mmol) in dichloromethane (100 mL) was stirred in the ice bath under moisture protection. Bocanhydride (5.45 g, 25 mmol) in dichloromethane (300 mL) was slowly added over a period of 6 h at 0°C. After stirring at room temperature over night in a nitrogen atmosphere, the solution was washed with brine (2x 100 mL) and water (2x 100 mL), dried over $MgSO_4$ and the solvent was removed at reduced

pressure. The crude oil was purified by column chromatography with silica gel and chloroform/methanol 6:1 containing 0.5 % aqueous, conc. ammonia solution, to give **12** as pale yellow oil (2.87 g, 15.24 mmol, 61 %).

^{1}H-NMR (300 MHz, $CDCl_3$): δ [ppm] = 3.21 (2 H, t, *J* = 6.4 Hz), 2.76 (3 H, s), 2.58 (2 H, t, *J* = 6.4 Hz), 2.32 (3 H, s), 1.36 (9 H, s), 1.12 (1 H, bs, NH); - **^{13}C-NMR** (150 MHz, $CDCl_3$): δ [ppm] = 155.8 (C_{quat}), 79.3 (C_{quat}), 49.6 (+), 48.3 (-), 36.2 (+), 34.6 (-), 28.3 (+); - **MS** (ESI-MS, CH_2Cl_2/MeOH + 10 mmol NH_4OAc): e/z (%) = 377.2 (12, $2MH^+$), 230.0 (26, MH^+ +MeCN), 189.0 (100, MH^+), 123.1 (5, MH^+ - C_4H_9); - **MW** = 188.27 g/mol; **MF** = $C_9H_{20}N_2O_2$

*3-Dimethylaminophenothiazin-5-ium triiodide (**10**) (literature known, improved procedure)*[iv]

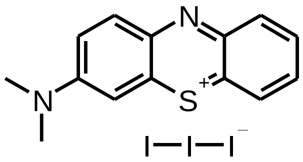

To a solution of phenothiazin-5-ium tetraiodide hydrate[v] (**9**) (7.23 g, 10 mmol) in dichloromethane (500 mL) was added solution of dimethylamine in methanol (2 M, 10.0 mL, 20 mmol) dropwise over 6 h. The reaction mixture was allowed to stand overnight at room temperature and the resultant precipitate was filtered off, washed with dichloromethane and allowed to air dry. The product was recrystallised from methanol to give **10** as dark-blue solid (3.30 g, 5.30 mmol, 53 %)

M.P. 144 – 145 °C; - **^{1}H-NMR** (300 MHz, DMSO-d6): δ [ppm] = 8.23 (1 H, dd, *J* = 8.0 & 1.6 Hz), 8.17 (1 H, dd, *J* = 8.0 & 1.6 Hz), 8.11 (1 H, d, *J* = 10 Hz), 8.04 (1 H, dd, *J* = 10 & 2.4 Hz), 7.99 (1 H, d, *J* = 2.4 Hz), 7.84 (2 H, m), 3.65 (3 H, s), 3.60 (3 H, s); - **^{13}C-NMR** (150 MHz, DMSO-d6): δ [ppm] = 156.0 (C_{quat}), 144.0 (C_{quat}), 139.7 (+), 139.5 (C_{quat}), 137.9 (C_{quat}), 134.5 (+), 133.2 (+), 129.7 (+), 126.2 (+), 125.9 (+), 125.8 (C_{quat}), 109.6 (+), 43.3 (+), 42.9 (+); - **IR** (neat): ν (cm^{-1}) = 2800 (bs), 1614 (s), 1585 (s), 1557 (s), 1492 (s), 1429 (m), 1404 (s), 1312 (m), 1245 (s), 1114 (s), 1073 (s), 880 (s), 829 (s), 765 (s); - **MS** (ESI-MS, CH_2Cl_2/MeOH + 10 mmol NH_4OAc): e/z (%) = 240.9 (100, M^+); - **MW** = 241.34 + 381.72 g/mol; **MF** = $C_{14}H_{13}N_2SI_3$

General procedure I: Synthesis of boc-protected methyleneblue derivatives

To a solution of 3-Dimethylaminophenothiazinium triiodide (**10**, 1.24 g, 2 mmol) in dichloromethane (500 mL) was added dropwise a solution of triethylamine (0.3 g, 0.4 mL, 3 mmol) in dichloromethane (50 mL). After stirring for 5 minutes the appropriate amine (6 mmol) in dichloromethane (250 mL) was added over a period of 2 h. The solution was stirred over night at room temperature and was then washed with water (3x 250 mL). The organic layer was dried over $MgSO_4$ and the solvent was evaporated at reduced pressure not exciding a water bath temperature of 40°C. The crude material was purified by repeated flash chromatography with silica gel using dichloromethane/ethanol 10:1 as the eluent.

*3-[(2-N-butyloxycarbonyl-2-aminoethyl)(methyl)amino]-7-(dimethylamino)phenothiazin-5-ium iodide (**14**)*[vi]

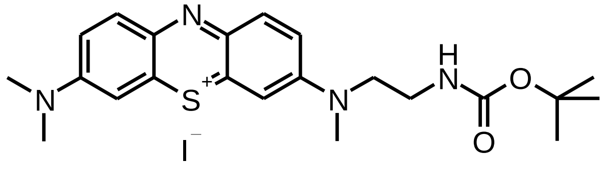

tert-butyl [2-(methylamino)ethyl]carbamate (**11**) (1.05 g, 6 mmol) was reacted to give 0.74 g bronze coloured glass (1.36 mmol, 68 %). $\mathbf{R_f}$ (DCM/EtOH 8:1 ~ 0.33)

^{1}H-NMR (300 MHz, $CDCl_3$): δ [ppm] = 7.78 – 7.89 (2 H, m), 7.12 – 7.41 (4 H, m), 6.12 (1 H, bs), 3.92 (2 H, t, *J* = 6.4 Hz), 3.52 (2 H, t, *J* = 6.4 Hz), 3.39 (3 H, s), 3.31 (6 H, s), 1.29 (9 H, s); - **^{13}C-NMR** (75 MHz, DMSO-d6): δ [ppm] = 155.7 (C_{quat}), 153.5 (C_{quat}), 137.6 (+), 137.4 (+), 134.9 (C_{quat}), 134.8 (C_{quat}), 133.4 (C_{quat}), 119.0 (+), 118.9 (+), 106.5 (+), 78.1 (C_{quat}), 52.2 (-), 46.2 (-), 41.1 (+), 39.6 (+), 27.8 (+); - **IR** (neat): ν (cm^{-1}) = 3330 (bs), 2976 (m), 2930 (m), 2906 (m), 2705 (m), 1699 (s), 1592 (s), 1546 (m), 1486 (s), 1438 (m), 1384 (s), 1314 (s), 1215 (s), 1161 (s), 1130 (s), 1070 (s), 1033 (s), 967 (m), 880 (s), 829 (s), 790 (s), 719 (m), 666 (m); - **MS** (ESI-MS, CH_2Cl_2/MeOH + 10 mmol NH_4OAc): e/z (%) = 413.0 (100, M^+); - **MW** = 413.57 +126.90 g/mol; **MF** = $C_{22}H_{29}N_4SO_2I$

*3-{methyl[2-(N-butyloxycarbonyl-methylammonio)ethyl]amino}-7-(dimethylamino)phenothiazin-5-ium iodide (**15**)*

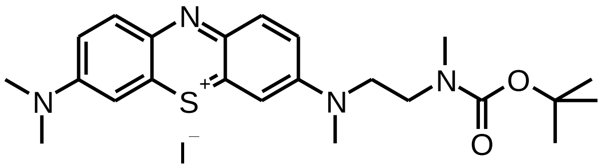

tert-butylmethyl[2-(methylamino)ethyl]carbamate (**12**) (1.12 g, 6 mmol) was reacted to give 0.69 g bronze coloured glass (1.25 mmol, 63 %). $\mathbf{R_f}$ (DCM/EtOH 8:1 ~ 0.35)

^{1}H-NMR (300 MHz, DMSO-d6): δ [ppm] = 7.76 – 7.88 (2 H, m), 7.42 – 7.56 (4 H, m), 3.81 (2 H, t, *J* = 6.4 Hz), 3.39 (3 H, s), 3.37 (6 H, s) 3.29 (3 H, s), 3.28 (2 H, t, *J* = 6.4 Hz), 1.27 (9 H, s); - **^{13}C-NMR** (75 MHz, DMSO-d6): δ [ppm] = 155.6 (C_{quat}), 153.9 (C_{quat}), 137.7 (+), 137.6 (+), 134.9 (C_{quat}), 134.8 (C_{quat}), 133.4 (C_{quat}), 119.1 (+), 118.9 (+), 106.8 (+), 77.8 (C_{quat}), 52.1 (-), 46.3 (-), 41.0 (+), 39.6 (+), 37.7 (+), 27.9 (+); - **IR** (neat): ν (cm^{-1}) = 3330 (bs), 2975 (m), 2930 (m), 2908 (m), 2701 (m), 1699 (s), 1591 (s), 1546 (m), 1485 (s), 1441 (m), 1384 (s), 1312 (s), 1214 (s), 1160 (s), 1129 (s), 1066 (s), 1031 (s), 967 (m), 871 (s), 829 (s), 789 (s), 719 (m), 665 (m); - **MS** (ESI-MS, CH_2Cl_2/MeOH + 10 mmol NH_4OAc): e/z (%) = 427.0 (100, M^+); - **MW** = 427.59 +126,90 g/mol; **MF** = $C_{23}H_{31}N_4SO_2I$

*3-[4-(tert-butoxycarbonyl)piperazin-1-yl]-7-(dimethylamino)phenothiazin-5-ium iodide (**16**)*

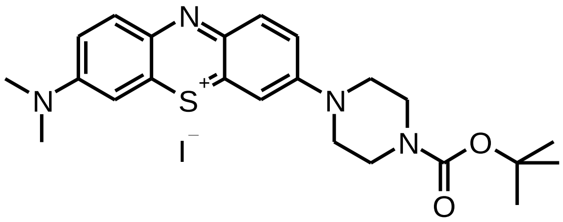

tert-butyl piperazine-1-carboxylate (1.11 g, 6 mmol) was reacted to give 0.78 g purple glimmering crystals (1.41 mmol, 71 %). $\mathbf{R_f}$ (DCM/EtOH 8:1 ~ 0.36)

^{1}H-NMR (300 MHz, DMSO-d6): δ [ppm] = 7.68 – 7.78 (2 H, m), 7.62 (1 H, m), 7.42 (1 H, m), 7.27 (1 H, m), 7.20 (1 H, m), 3.83 (4 H, m), 3.62 (4 H, m), 3.37 (6 H, s), 1.41 (9 H, s); - **^{13}C-NMR** (75 MHz, DMSO-d6): δ [ppm] = 154.4 (C_{quat}), 154.0 (C_{quat}), 153.1 (C_{quat}), 138.6

(+), 138.4 (+), 135.7 (C_{quat}), 135.1 (C_{quat}), 133.4 (C_{quat}), 119.4 (+), 118.9 (+), 107.5 (+), 107.2 (+), 106.6 (+), 80.6 (C_{quat}), 47.7 (-), 47.2 (-), 42.4 (+), 28.4 (+); - **IR** (neat): ν (cm^{-1}) = 3443 (bs), 2973 (w), 2926 (w), 2867 (w), 2704 (w), 2185 (w), 1683 (s), 1593 (s), 1487 (m), 1448 (m), 1388 (s), 1332 (s), 1219 (s), 1121 (s), 1080 (m), 1041 (m), 991 (m), 882 (s), 836 (m), 789 (m), 724 (s); - **MS** (ESI-MS, CH_2Cl_2/MeOH + 10 mmol NH_4OAc): e/z (%) = 425.2 (100, M^+); - **MW** = 425.58 +126.90 g/mol; **MF** = $C_{23}H_{29}N_4SO_2I$

General procedure II: Deprotection of boc-protected methyleneblue derivatives

a) Deprotection

The boc-protected methylenblue derivative (0.5 mmol) was dissolved in dichloromethane (4 mL). TFA (285 mg, 0.2 mL, 2.5 mmol) in dichloromethane (2 mL, 10% TFA) was added dropwise and the reaction mixture was stirred for 5h at room temperature. The solution was transferred to four blue caps, the product was precipitated by addition of diethylether (13.5 mL per tube) and centrifuged. The solution was decanted off the precipitate, it was resuspended in diethylether (15 mL per tube) and centrifuged again. The solvent was decanted off and the residue was dried at reduced pressure without heating.

b) Ion exchange

A small column was packed with ion exchanger (Amberlite IRA-958). The resin was rinsed with acidic sodium chloride solution (10 % aqueous NaCl cont. 0.1 % HCl, 100 mL) and conditioned with dilute hydrochloric acid (0.1 %).

The TFA salt from the former step was dissolved in double distilled water (5 mL) and was passed through the column (height 10 cm, diameter 1 cm) of anion exchanger (Amberlite IRA-958) eluting with water (40 mL). The aqueous solution was lyophilized to give the product as dark blue solid.

*3-[(2-Ammoniumethyl)(methyl)amino]-7-(dimethylamino)phenothiazin-5-ium dichloride (literature known compound) (**1**)*[vi]

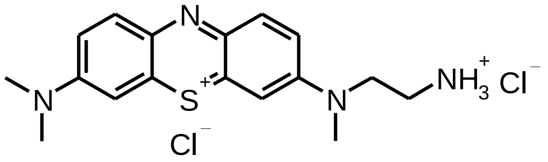

Conversion of compound **14** by general procedure II gives the desired product **1** as dark blue powder (185 mg, 0.476 mmol, 95 %). $\mathbf{R_f}$ ($CHCl_3$/MeOH 4:1 ~ 0.1)

^{1}**H-NMR** (300 MHz, DMSO-d6): δ [ppm] = 7.72 – 7.91 (2 H, m), 7.23 – 7.51 (4 H, m), 3.95 (2 H, m), 3.67 (6 H, s), 3.24 (3 H, s), 3.08 (2 H, m); - **MS** (ESI-MS, CH_2Cl_2/MeOH + 10 mmol NH_4OAc): e/z (%) = 313.1 (100, M^+), 157.6 (18, $(M + 2H^+)^{2+}$), 148.6 (51, $(M\text{-}NH_3 + 2H^+)^{2+}$); - **MW** =314.46 +2x 35.45 g/mol; **MF** = $C_{17}H_{22}N_4SCl_2$

*3-{methyl[2-(methylammonio)ethyl]amino}-7-(dimethylamino)phenothiazin-5-ium dichloride (**2**)*

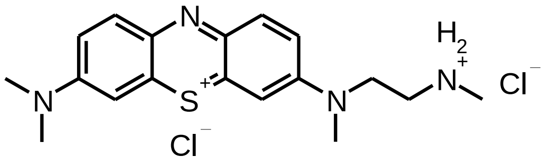

Deprotection of **15** and ion exchange after general procedure II gives product **2** as dark blue powder (185 mg, 0.481 mmol, 96 %). $\mathbf{R_f}$ ($CHCl_3$/MeOH 4:1 ~ 0.1)

^{1}**H-NMR** (300 MHz, DMSO-d6): δ [ppm] = 7.83 – 7.96 (2 H, m), 7.41 – 7.63 (4 H, m), 4.03 (2 H, m), 3.65 (6 H, s), 3.31 (3 H, s), 3.19 (2 H, m), 2.63 (3 H, s); - **MS** (ESI-MS, CH_2Cl_2/MeOH + 10 mmol NH_4OAc): e/z (%) = 327.2 (100, M^+), 164.1 (43, $(M + 2H^+)^{2+}$), 155.6 (6, $(M\text{-}NH_3 + 2H^+)^{2+}$); - **MW** = 328.48 +2x 35.45 g/mol; **MF** = $C_{18}H_{24}N_4SCl_2$

*3-(piperazin-4-ium-1-yl)-7-(dimethylamino)phenothiazin-5-ium dichloride (**3**)*

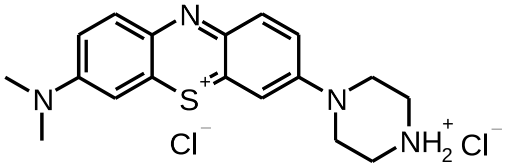

Deprotection of **15** and ion exchange after general procedure II gives product **2** as dark blue powder (184 mg, 0.489 mmol, 98 %). $\mathbf{R_f}$ ($CHCl_3$/MeOH 4:1 ~ 0.1)

^{1}**H-NMR** (300 MHz, DMSO-d6): δ [ppm] = 7.84 – 7.98 (2 H, m), 7.46 – 7.67 (4 H, m), 3.64 (6 H, s), 3.36 (4 H, m), 3.24 (4 H, m); - **MS** (ESI-MS, CH_2Cl_2/MeOH + 10 mmol NH_4OAc): e/z (%) = 325.1 (89, M^+), 163.1 (100, $(M +2H^+)^{2+}$), 154.5 (3, $(M\text{-}NH_3 +2H^+)^{2+}$); - **MW** = 326.47 +2x 35.45 g/mol; **MF** = $C_{18}H_{22}N_4SCl_2$

General procedure III: Synthesis of asymmetric methyleneblue derivatives

To a solution of 3-Dimethylaminophenothiazinium triiodide (**10**, 1.24 g, 2 mmol) in dichloromethane (360 mL) was added dropwise a solution of the appropriate amine (12 mmol) in dichloromethane (40 mL) over a period of 1 h. The solution was stirred over night at room temperature and was then washed with water (1x 200 mL). The organic layer was dried over $MgSO_4$ and the solvent was evaporated at reduced pressure not exciding a water bath temperature of 40°C. The crude material was dissolved in dichloromethane (10 mL) and precipitated by addition of diethylether (50 mL). The precipitate was settled by the aid of a centrifuge, the supernatant was decanted off and the dark blue residue was purified by repeated flash chromatography with silica gel.

General procedure IV: Ion exchange protocol for methyleneblue derivatives

The column was packed with ion exchanger (Amberlite IRA-958). The resin was rinsed with acidic sodium chloride solution (10 % aqueous NaCl cont. 0.1 % HCl, 100 mL) and conditioned with dilute hydrochloric acid (0.1 %).

The methyleneblue derivative (0.5 mmol) was dissolved in hydrochloric acid (1M, 10 mL) and lyophilized. A solution of this mixed salt was dissolved in double distilled water (6 mL) and was slowly passed through a column (height 10 cm, diameter 1 cm; transferred with 4 mL dilute hydrochloric acid 0.1%) of the conditioned anion exchanger (Amberlite IRA-958) eluting with dilute hydrochloric acid (40 mL, 0.1 %). The aqueous solution was lyophilized to give the product as dark blue solid.

*3-[(2-Hydroxyethyl)(methyl)amino]-7-(dimethylamino)phenothiazin-5-ium chloride (**4**)*

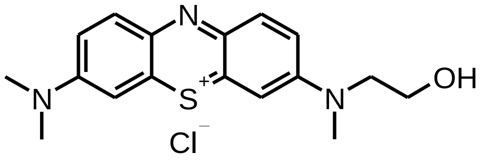

2-N-Methyl-aminoethanol (0.90 g, 0.96 mL, 12 mmol) was reacted and the crude material was purified with dichloromethane/ethanol 8:1 and dichloromethane/methanol 5:1 to give 0.38 g of a dark blue, purple glimmering glass (0.85 mmol, 42 %). After ion exchange 0.33 g of a dark blue, purple glimmering powder is isolated. **R_f** (DCM/EtOH 8:1 ~ 0.16)

^{1}H-NMR (300 MHz, DMSO-d6): δ [ppm] = 7.94 (2 H, m), 7.59 (2 H, m), 7.51 (2 H, m), 5.06 (1 H, m), 4.09 (3 H, s), 3.88 (2 H, m), 3.69 (2 H, m), 3.38 (6 H, s); - **^{13}C-NMR** (75 MHz, DMSO-d6): δ [ppm] = 154.1 (C_{quat}), 153.8 (C_{quat}), 137.6 (+), 137.5 (+), 134.6 (C_{quat}), 134.4 (C_{quat}), 133.6 (C_{quat}), 119.1 (+), 118.8 (+), 106.7 (+), 106.5 (+), 58.4 (-), 54.3 (-), 41.1 (+), 40.9 (+); - **IR** (neat): ν (cm^{-1}) = 3384 (bs), 3045 (w), 2913 (w), 2869 (w), 2705 (w), 1592 (s), 1545 (m), 1538 (m), 1487 (m), 1443 (m), 1389 (s), 1329 (s), 1248 (m), 1224 (m), 1180 (m), 1142 (m), 1063 (s), 975 (m), 880 (m), 815 (m), 665 (m); - **MS** (ESI-MS, CH_2Cl_2/MeOH + 10 mmol NH_4OAc): e/z (%) = 314.1 (100, M^+); - **MW** = 314.43 + 35.45 g/mol; **MF** = $C_{17}H_{20}N_3SOCl_2$

*3-(morpholin-4-yl)-7-(dimethylamino)phenothiazin-5-ium chloride (**5**)*[vii]

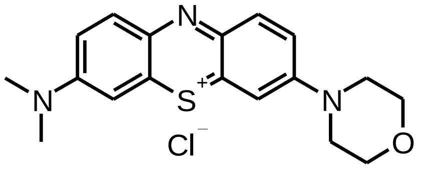

Morpholine (1.05 g, 1.05 mL, 12 mmol) was reacted and the crude material was purified with dichloromethane/ethanol 8:1 and recrystallised from ethanol to give 0.49 g bronze coloured crystal plates (1.09 mmol, 54 %). After ion exchange 0.38 g of a dark blue, purple glimmering powder is isolated. $\mathbf{R_f}$ (DCM/EtOH 8:1 ~ 0.21)

^{1}H-NMR (600 MHz, DMSO-d6): δ [ppm] = 7.94 (2 H, dd, *J* = 13.2 & 5.4 Hz), 7.69 (1 H, d, *J* = 5.4 Hz), 7.63 (1 H, dd, *J* = 13.2 & 5.4 Hz), 7.57 (1 H, d, *J* = 5.4 Hz), 7.55 (1 H, dd, *J* = 13.2 & 5.4 Hz), 3.83 (4 H, t, *J* = 5.6 Hz), 3.78 (4 H, t, *J* = 5.6 Hz), 3.39 (6 H, s); - **^{13}C-NMR** (150 MHz, DMSO-d6): δ [ppm] = 154.1 (C_{quat}), 153.1 (C_{quat}), 138.1 (+), 137.8 (+), 135.9 (C_{quat}), 134.9 (C_{quat}), 134.2 (C_{quat}), 133.5 (C_{quat}), 119.9 (+), 118.6 (+), 107.1 (+), 107.0 (+), 65.9 (-), 54.9 (+), 47.5 (-), 41.3 (+); - **IR** (neat): ν (cm^{-1}) = 3441 (bs), 3039 (w), 2885 (w), 2849 (w), 2700 (w), 1592 (s), 1485 (m), 1446 (m), 1391 (s), 1351 (s), 1307 (m), 1236 (s), 1173 (m), 1141 (m), 1112 (m), 1041 (m), 945 (m), 883 (s), 819 (m), 628 (m); - **MS** (ESI-MS, CH_2Cl_2/MeOH + 10 mmol NH_4OAc): e/z (%) = 326.1 (100, M^+); - **MW** = 326.44 + 35.45 g/mol; **MF** = $C_{18}H_{20}N_3SOCl$

*3-(4-methylpiperazin-4-ium-1-yl)-7-(dimethylamino)phenothiazin-5-ium dichloride (**6**)*

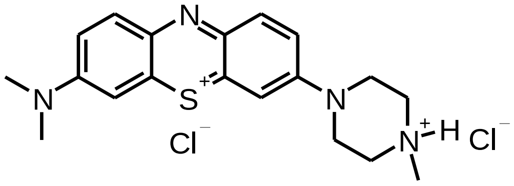

1-N-Methyl-piperazin (1.2 g, 1.34 mL, 12 mmol) was reacted and the crude material was purified with dichloromethane/methanol 8:1 → 5:1 and dichloromethane/methanol 5:1 to give 0.20 g of a dark blue, purple glimmering glass (0.43 mmol, 21 %). After ion exchange 0.17 g of a dark blue, purple glimmering glass is isolated. $\mathbf{R_f}$ (DCM/EtOH 8:1 ~ 0.02); $\mathbf{R_f}$ ($CHCl_3$/MeOH 4:1 ~ 0.15)

^{1}**H-NMR** (300 MHz, DMSO-d6): δ [ppm] = 8.03 (2 H, dd, *J* = 13.2 & 5.6 Hz), 7.88 (1 H, m), 7.62 (3 H, m), 4.52 (2 H, m), 3.83 (2 H, m), 3.55 (2 H, m), 2.81 (6 H, s), 3.21 (2 H, m), 2.81 (3 H, s); - ^{13}**C-NMR** (75 MHz, DMSO-d6): δ [ppm] = 154.1 (C_{quat}), 153.1 (C_{quat}), 138.1 (+), 137.8 (+), 135.9 (C_{quat}), 134.9 (C_{quat}), 134.2 (C_{quat}), 133.5 (C_{quat}), 119.9 (+), 118.6 (+), 107.1 (+), 107.0 (+), 65.9 (-), 54.9 (+), 47.5 (-), 41.3 (+); - **IR** (neat): ν (cm^{-1}) = 3423 (bs), 3032 (w), 2941 (w), 2853 (w), 2807 (w), 2686 (w), 1593 (s), 1517 (m), 1484 (m), 1452 (m), 1394 (s), 1353 (s), 1286 (m), 1240 (s), 1221 (s), 1179 (m), 1131 (s), 1081 (m), 1042 (m), 996 (s), 947 (m), 881 (s), 823 (m), 813 (m), 631 (m); - **MS** (ESI-MS, CH_2Cl_2/MeOH + 10 mmol NH_4OAc): e/z (%) = 339.2 (100, M^+), 170.1 (86, $(M +2H^+)^{2+}$); **MW** = 340.49 +2x 35.45 g/mol; **MF** = $C_{19}H_{24}N_4SCl_2$

Selected NMR spectra of prepared compounds

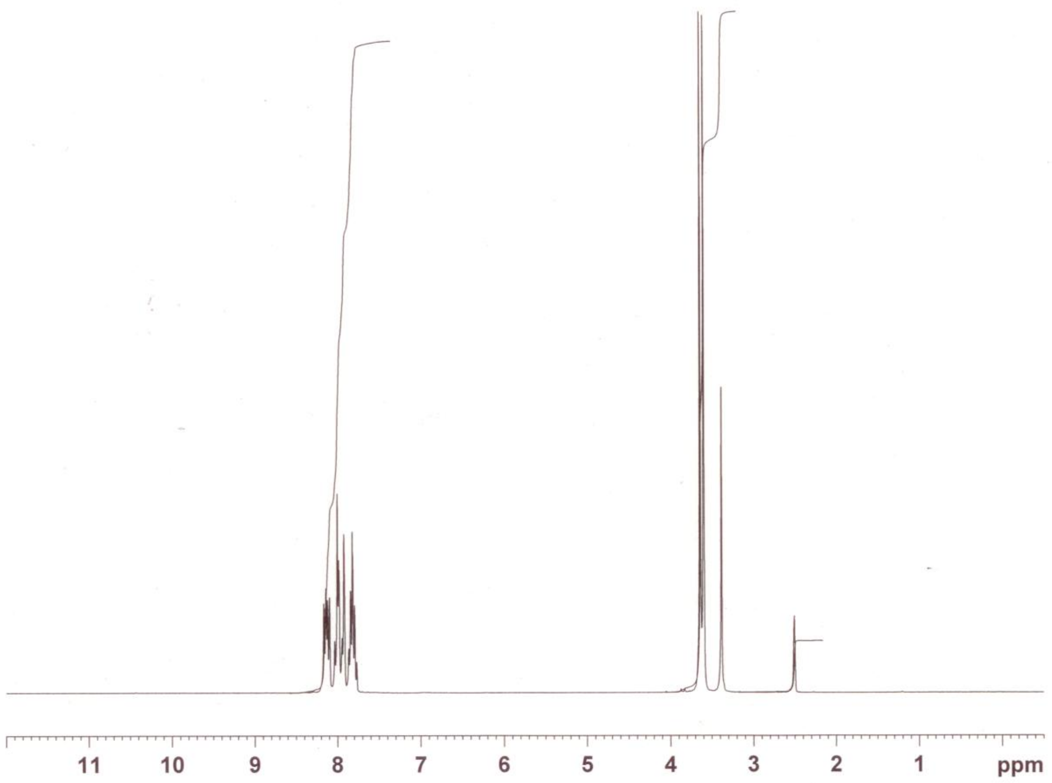

Figure S-2: ^{1}H-NMR spectrum of compound **10**

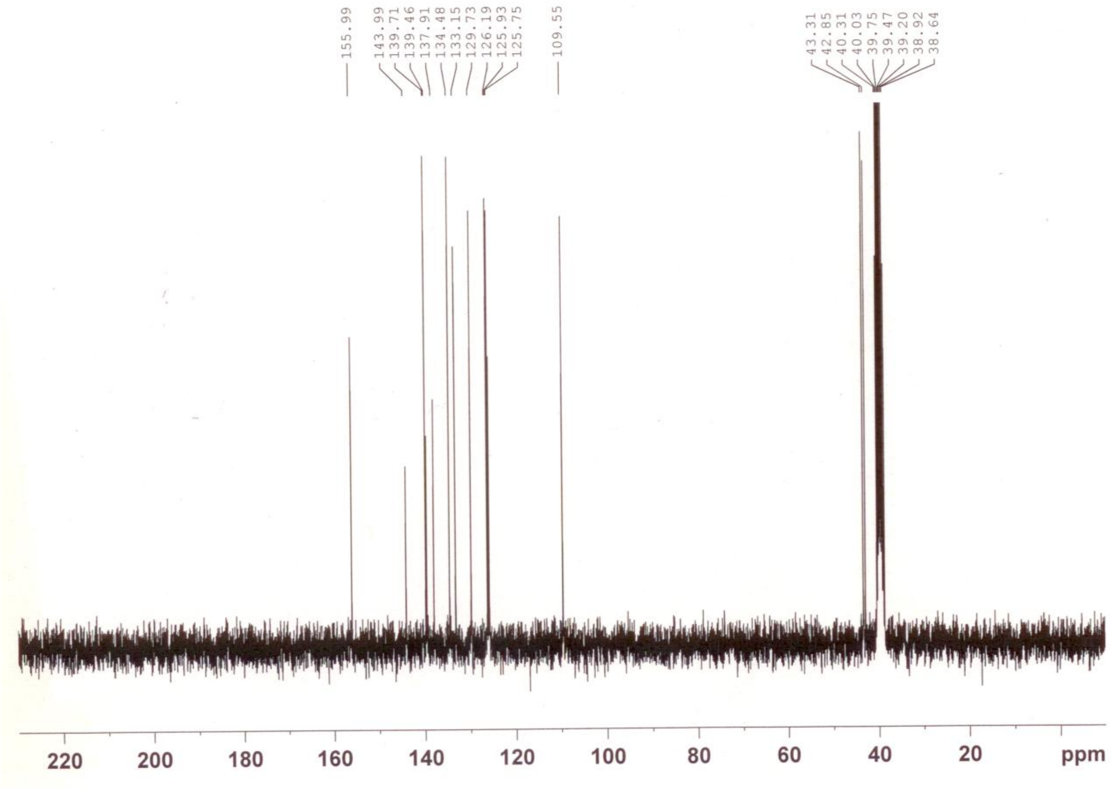

Figure S-3: ^{13}C-NMR spectrum of compound **10**

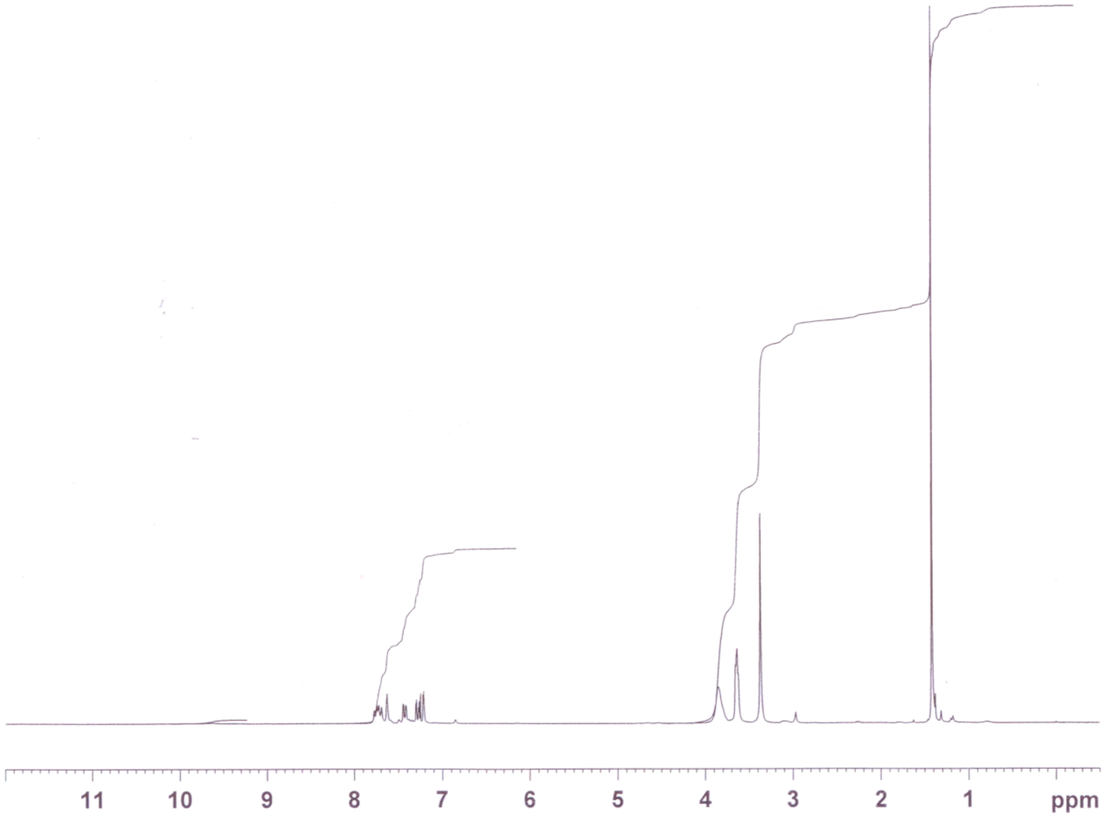

Figure S-4: ^{1}H-NMR spectrum of compound **16**

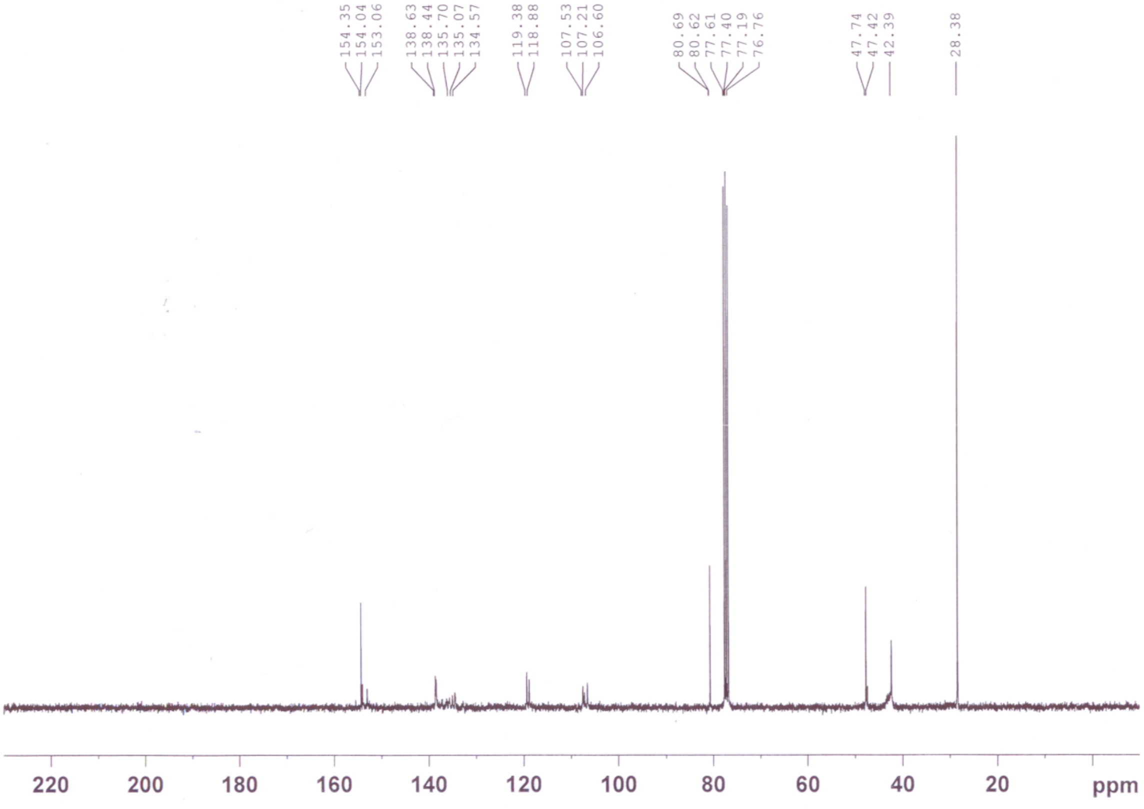

Figure S-5: ^{13}C-NMR spectrum of compound **16**

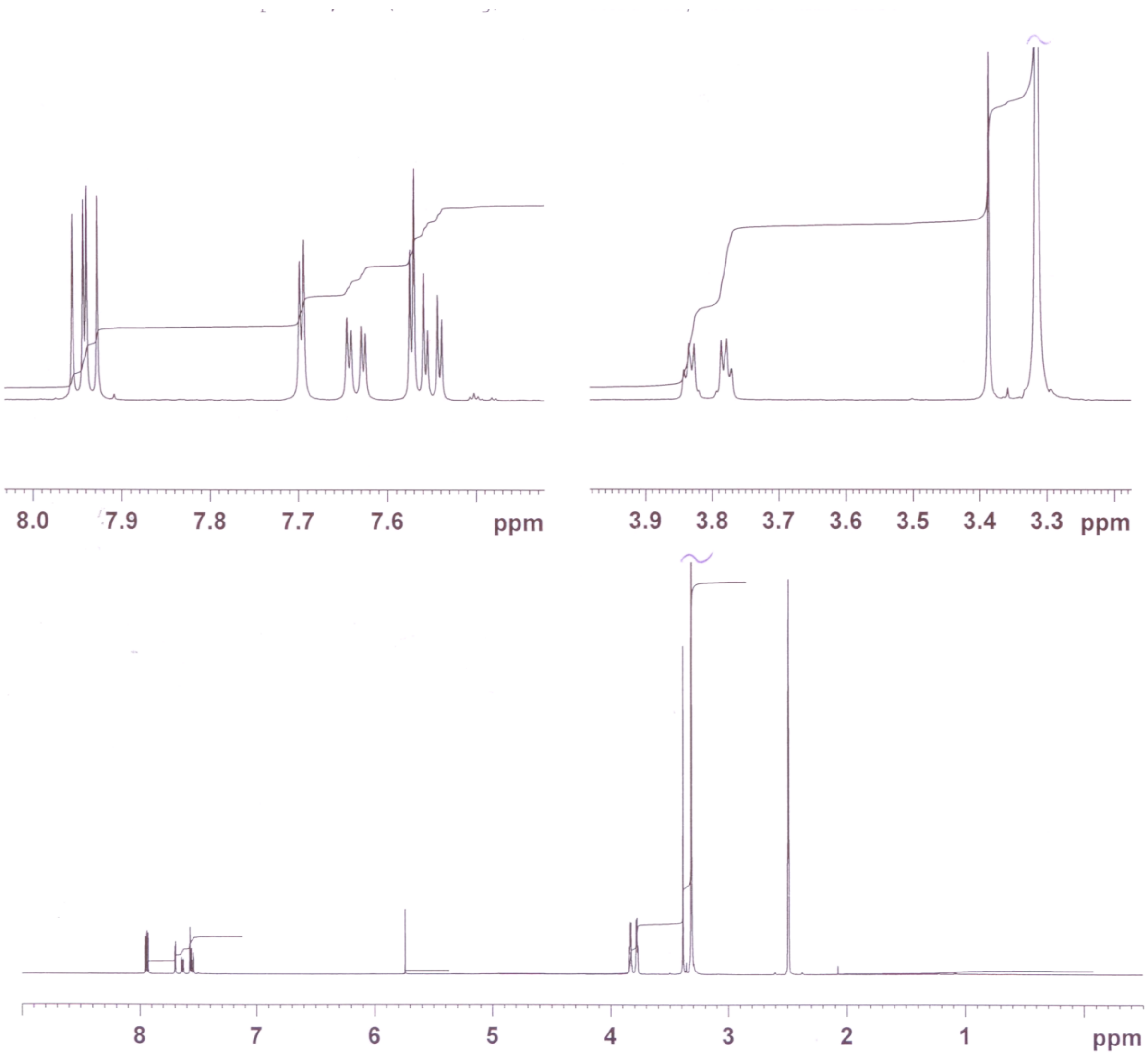

Figure S-6: ^{1}H-NMR spectra of compound **5**

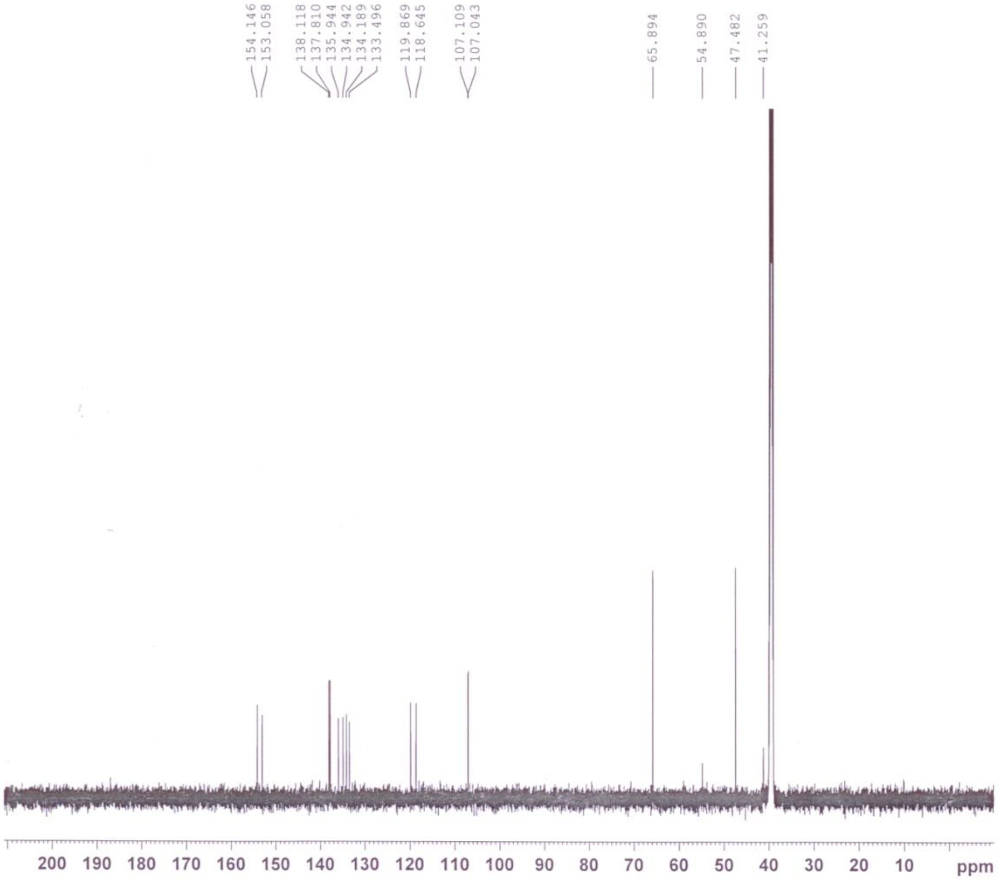

Figure S-7: ^{13}C-NMR spectrum of compound **5**

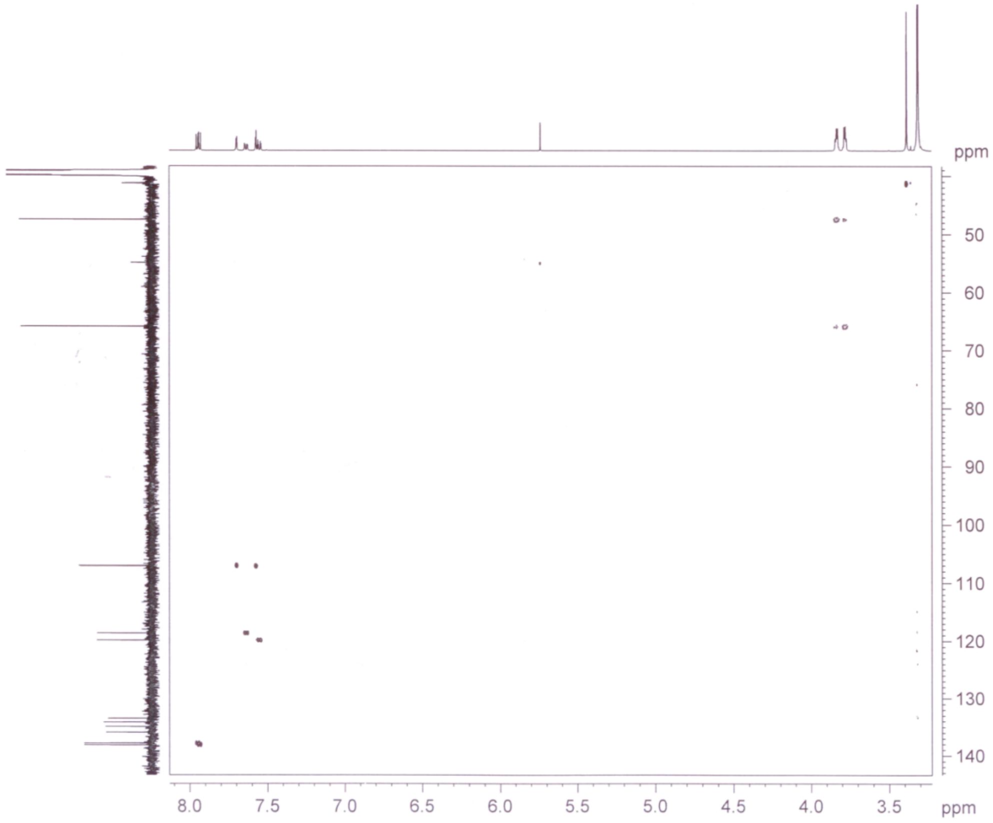

Figure S-8: HSQC spectrum of compound **5**

Fotographs of selected compounds

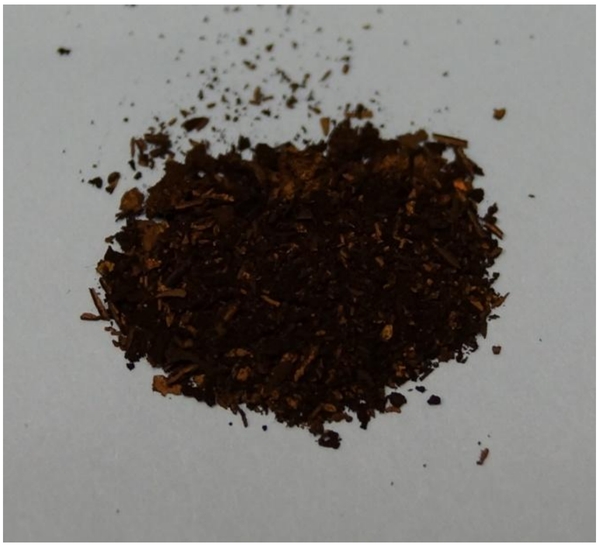

Figure S-9: Compound **15**

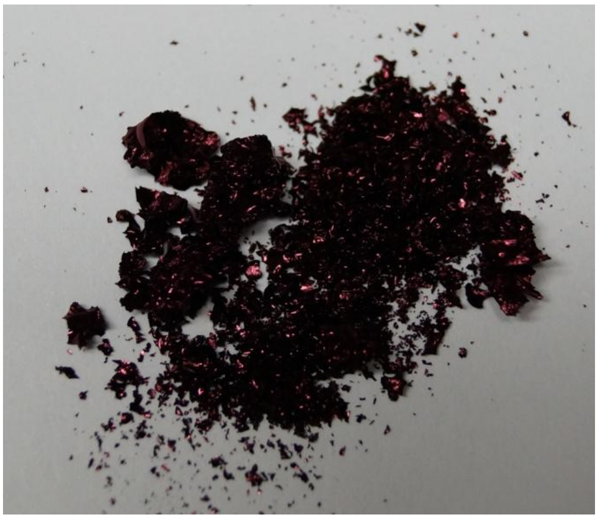

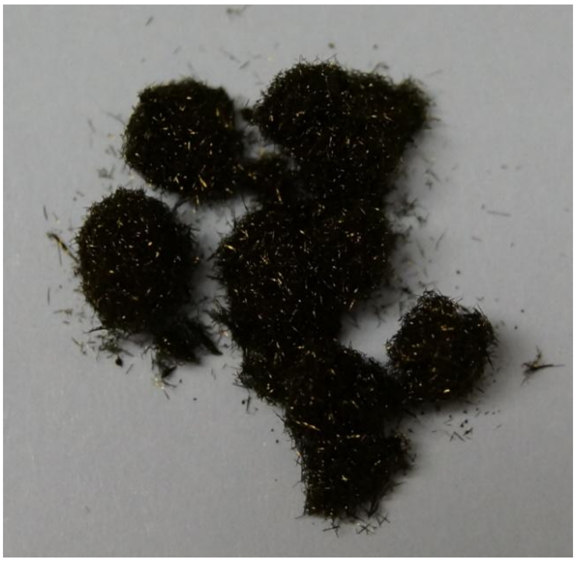

Figure S-10: Crystalline MB derivatives, compound **16** (left) and compound **6-I** (right)

Photophysical Characterisation

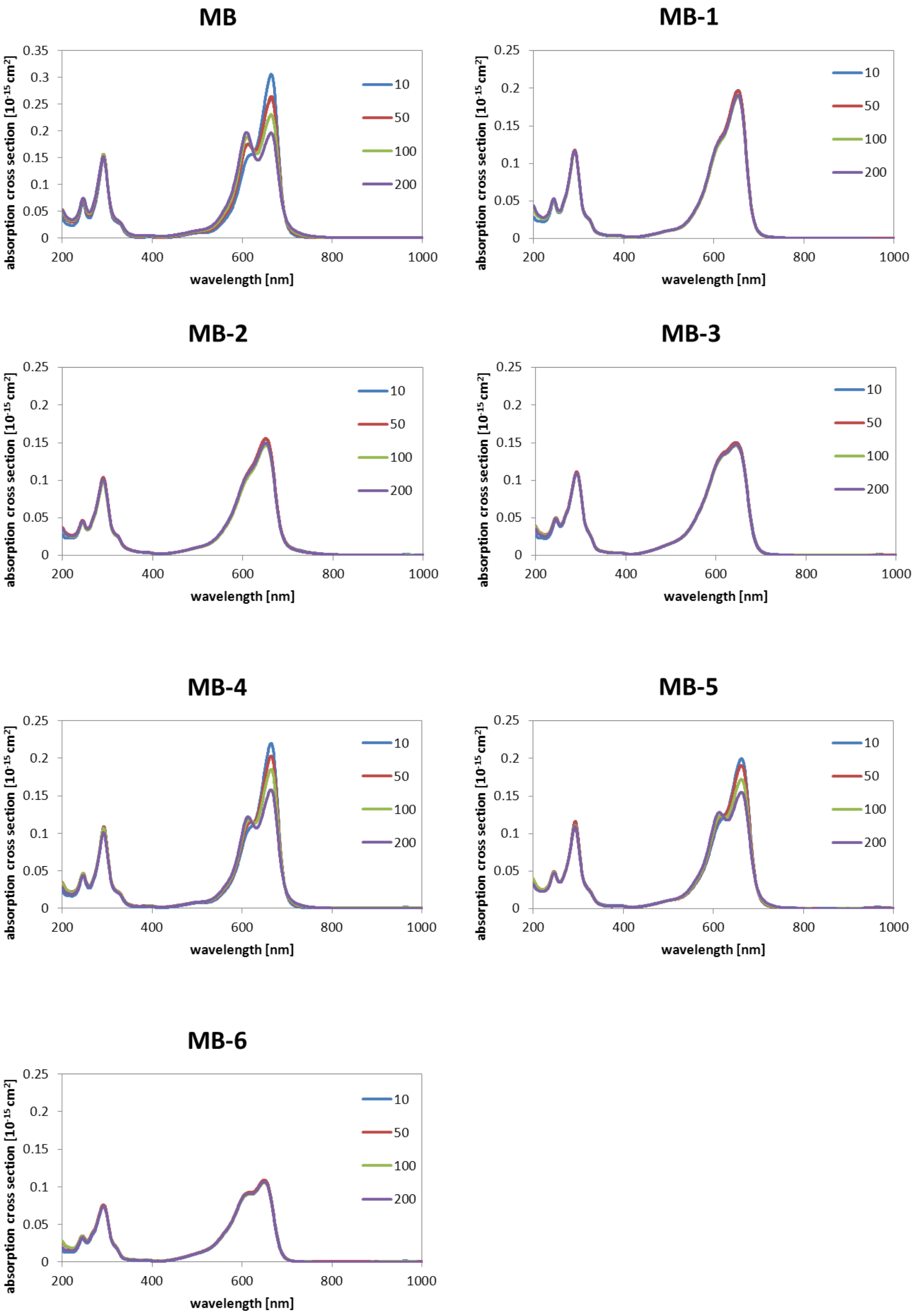

Figure S-11: Absorption spectra of MB and its derivatives within a concentration range of 10 – 200 µM in H_2O; the graphs show dimerisation for MB, MB-4 and MB-5.

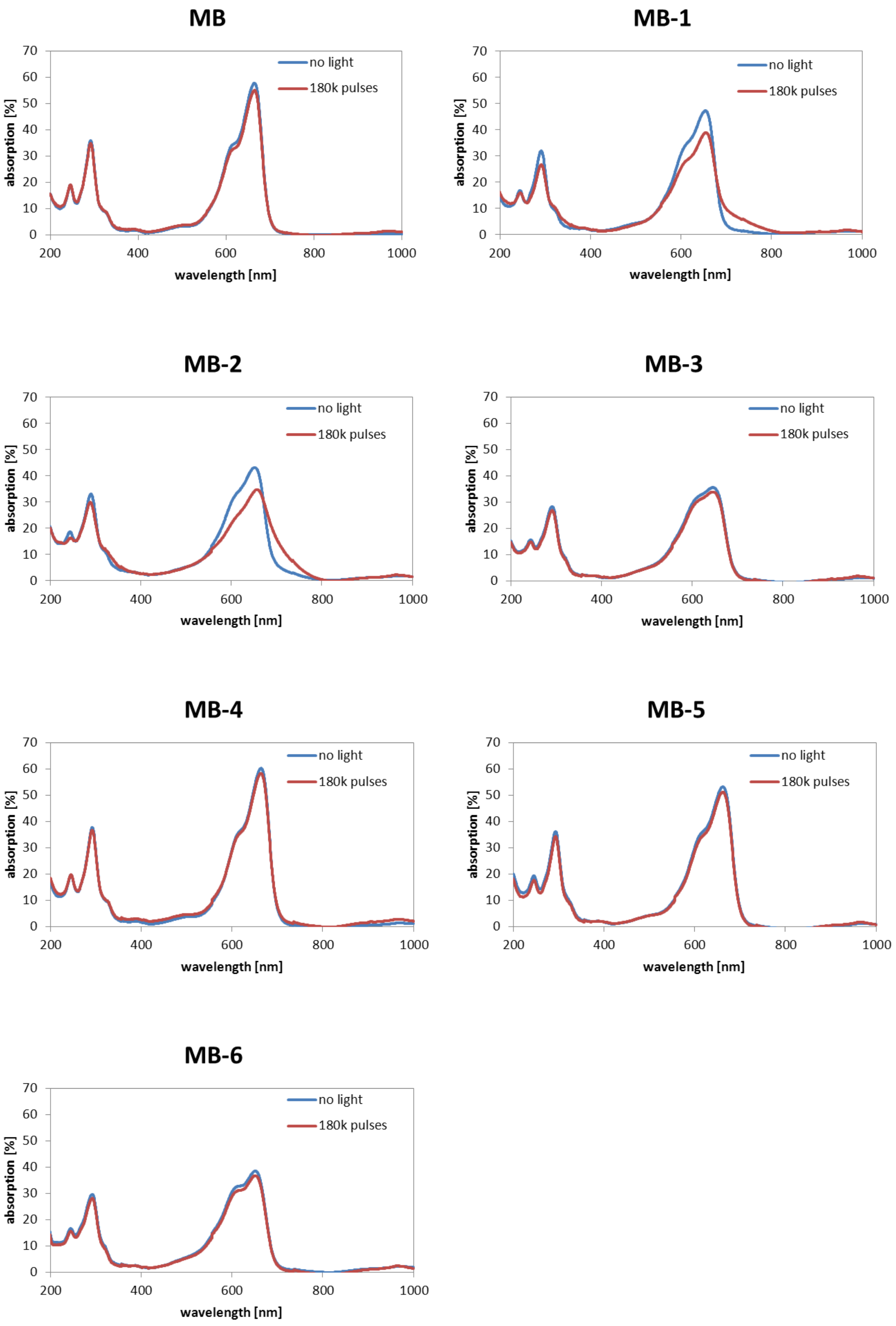

Figure S-12: Photostability measurements in a quartz cuvette with an irradiation at 600 nm with 180000 laser pulses; only MB-1 and MB-2 show a significant decrease in the absorption in the visible and in the UV range.

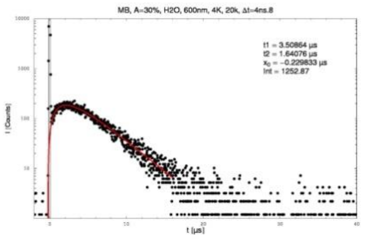
MB, A=30%, H2O, 600nm, 4K, 20k, Δt=4ns.8
t1 = 3.50864 µs
t2 = 1.64076 µs
x_0 = −0.229833 µs
Int = 1252.87
I [Counts]
t [µs]

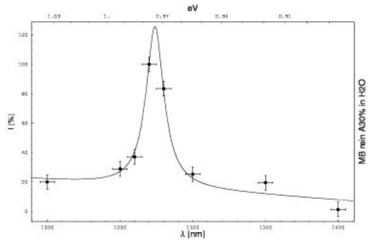
eV
I [%]
λ [nm]
MB rein A30% in H2O

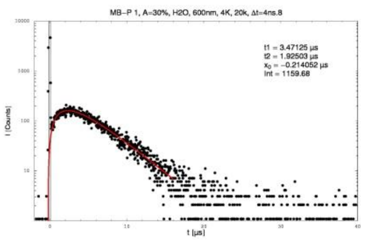
MB−P 1, A=30%, H2O, 600nm, 4K, 20k, Δt=4ns.8
t1 = 3.47125 µs
t2 = 1.92503 µs
x_0 = −0.214052 µs
Int = 1159.68
I [Counts]
t [µs]

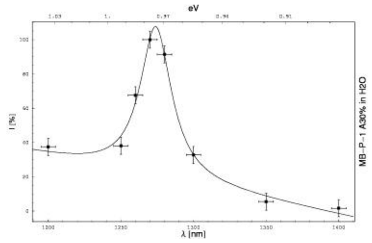
eV
I [%]
λ [nm]
MB−P−1 A30% in H2O

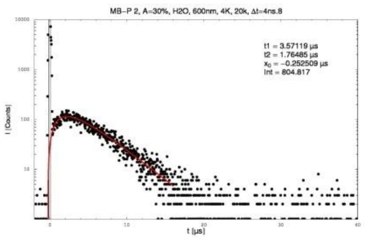
MB−P 2, A=30%, H2O, 600nm, 4K, 20k, Δt=4ns.8
t1 = 3.57119 µs
t2 = 1.76485 µs
x_0 = −0.252509 µs
Int = 804.817
I [Counts]
t [µs]

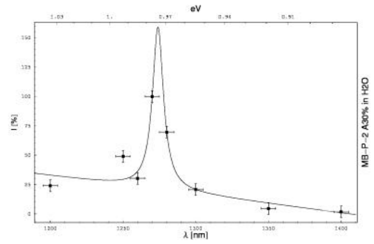
eV
I [%]
λ [nm]
MB−P−2 A30% in H2O

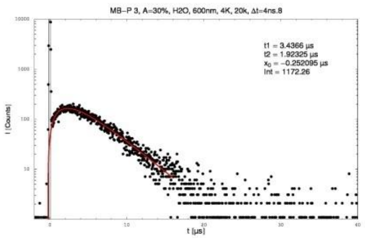
MB−P 3, A=30%, H2O, 600nm, 4K, 20k, Δt=4ns.8
t1 = 3.4366 µs
t2 = 1.92325 µs
x_0 = −0.252095 µs
Int = 1172.26
I [Counts]
t [µs]

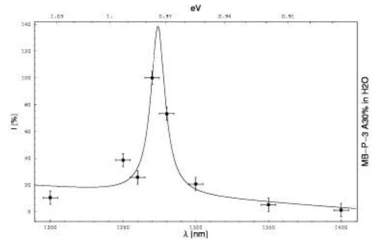
eV
I [%]
λ [nm]
MB−P−3 A30% in H2O

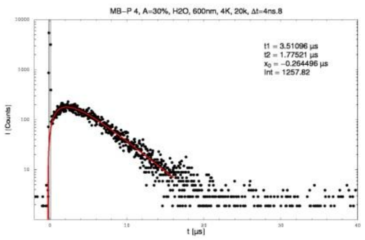
MB−P 4, A=30%, H2O, 600nm, 4K, 20k, Δt=4ns.8
t1 = 3.51096 µs
t2 = 1.77521 µs
x_0 = −0.264496 µs
Int = 1257.82
I [Counts]
t [µs]

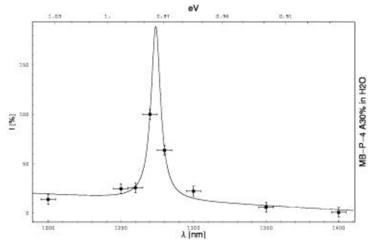
eV
I [%]
λ [nm]
MB−P−4 A30% in H2O

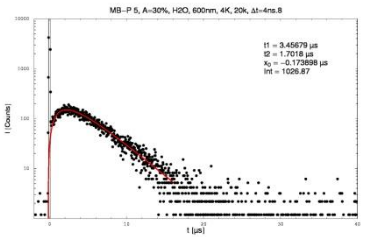
MB−P 5, A=30%, H2O, 600nm, 4K, 20k, Δt=4ns.8
t1 = 3.45679 µs
t2 = 1.7018 µs
x_0 = −0.173898 µs
Int = 1026.87
I [Counts]
t [µs]

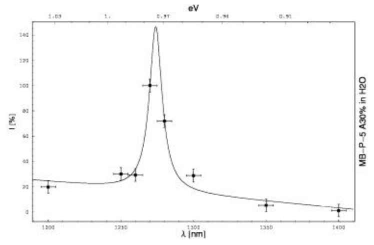
eV
I [%]
λ [nm]
MB−P−5 A30% in H2O

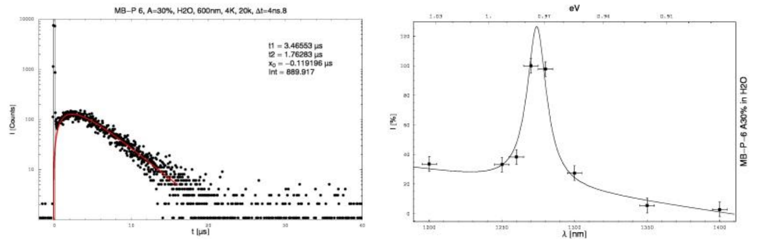

Figure S-13: Time- and spectrally resolved singlet oxygen luminescence of MB and its derivatives in air saturated H_2O at 25°C. Singlet oxygen is generated and detected at 1275 nm with a decay time ≈3.5 μs for all derivatives.

Antimicrobial Acitivity Data

MB-1

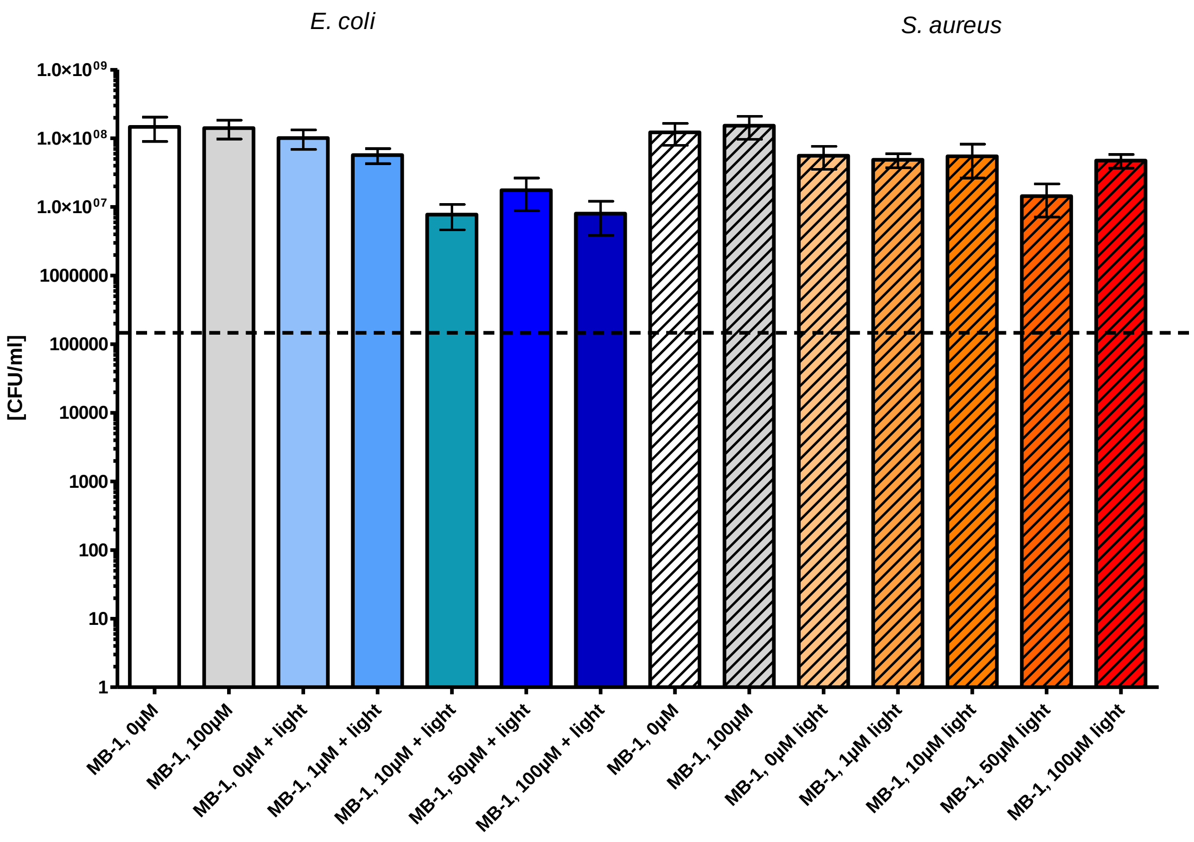

Figure S-14: Photodynamic inactivation of *E. coli* and *S. aureus* by methylene blue derivative MB-1.

Photodynamic treatment was performed using different concentrations of MB-1 with and without illumination (30 J cm^{-2}). Surviving colonies were counted 24 h later. Black dashed line corresponds to a reduction of 3 log_{10} steps in bacteria numbers (99.9% killing efficacy). White and grey column: controls without illumination. Coloured columns: MB-1 + light activation, blue: *E. coli*; crosshatched: *S. aureus*. (n=3 experiments: mean values ± standard deviation)

MB-2

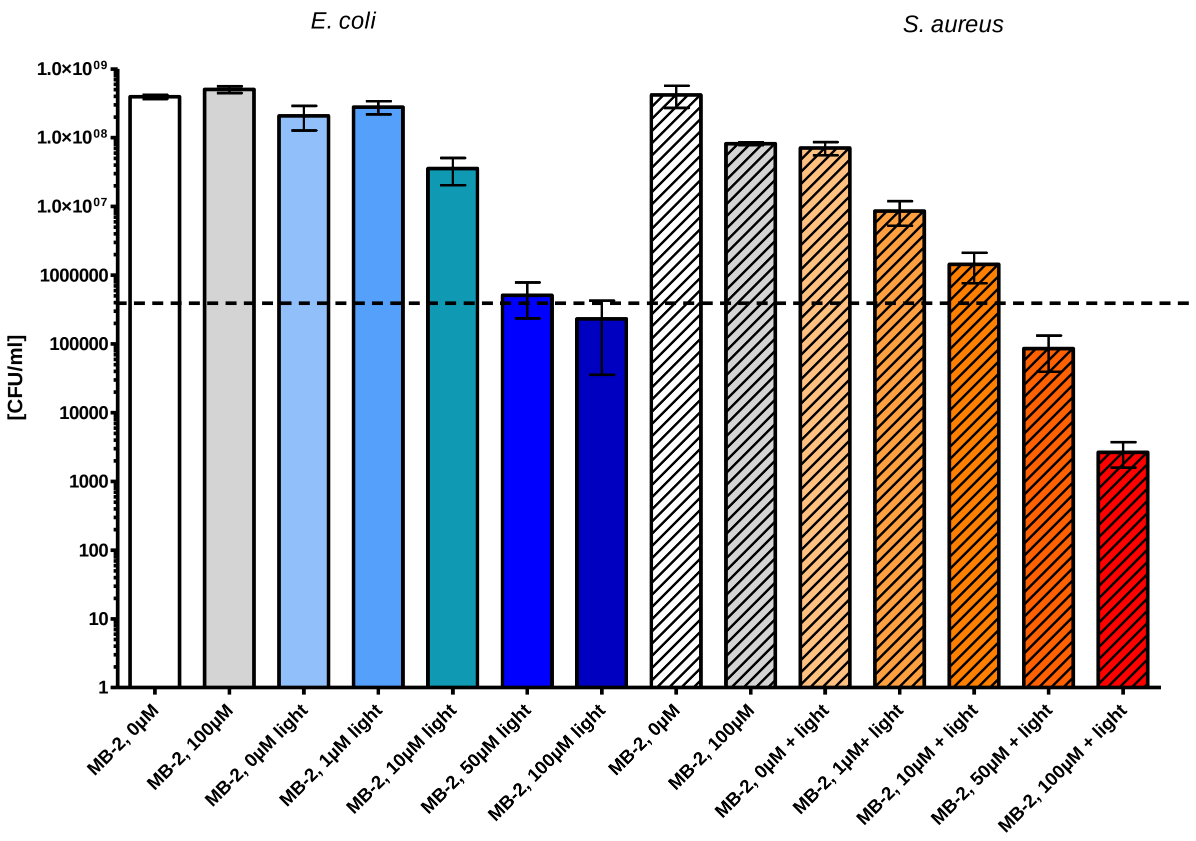

Figure S-15: Photodynamic inactivation of *E. coli* and *S. aureus* by methylene blue derivative MB-2.

Photodynamic treatment was performed using different concentrations of MB-2 with and without illumination (30 J cm^{-2}). Surviving colonies were counted 24 h later. Black dashed line corresponds to a reduction of 3 log_{10} steps in bacteria numbers (99.9% killing efficacy). White and grey column: controls without illumination. Coloured columns: MB-2 + light activation, blue: *E. coli*; crosshatched: *S. aureus*. (n=3 experiments: mean values ± standard deviation)

MB-3

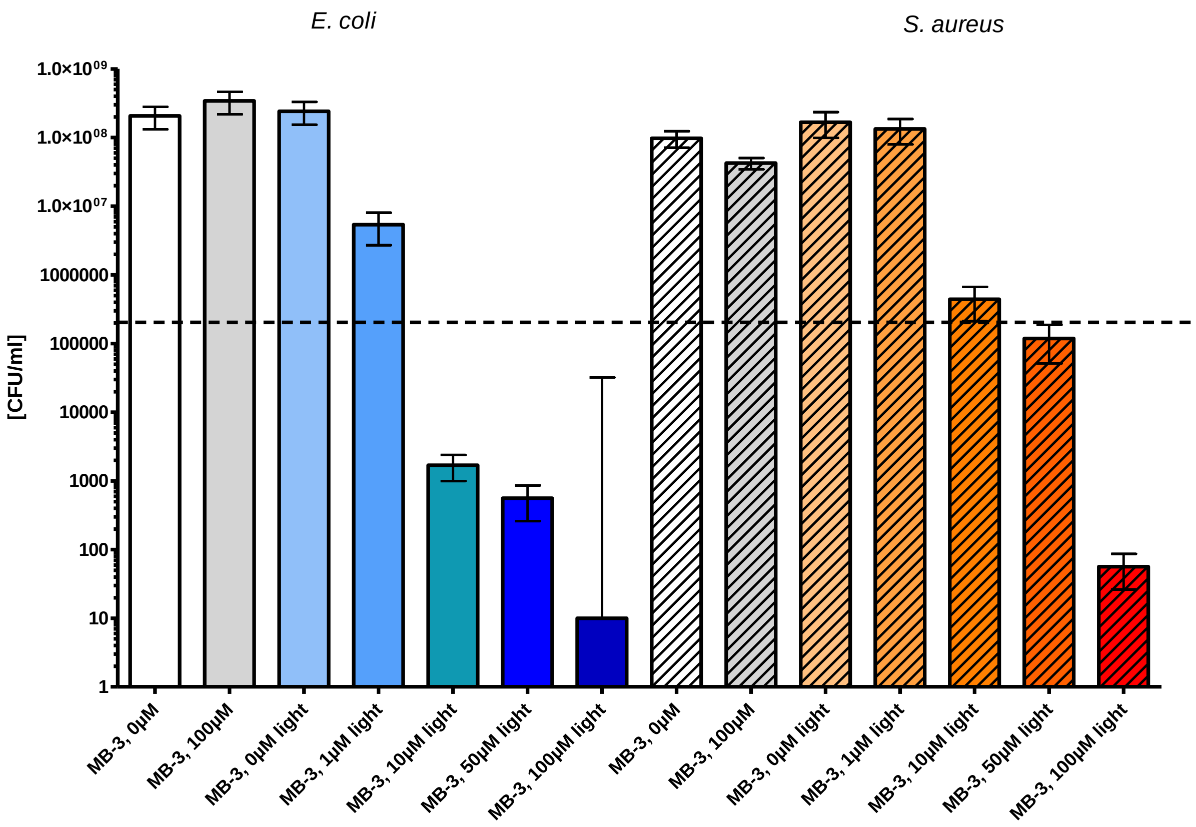

Figure S-16: Photodynamic inactivation of *E. coli* and *S. aureus* by methylene blue derivative MB-3.

Photodynamic treatment was performed using different concentrations of MB-3 with and without illumination (30 J cm^{-2}). Surviving colonies were counted 24 h later. Black dashed line corresponds to a reduction of 3 log_{10} steps in bacteria numbers (99.9% killing efficacy). White and grey column: controls without illumination. Coloured columns: MB-3 + light activation, blue: *E. coli*; crosshatched: *S. aureus*. (n=3 experiments: mean values ± standard deviation)

MB-4

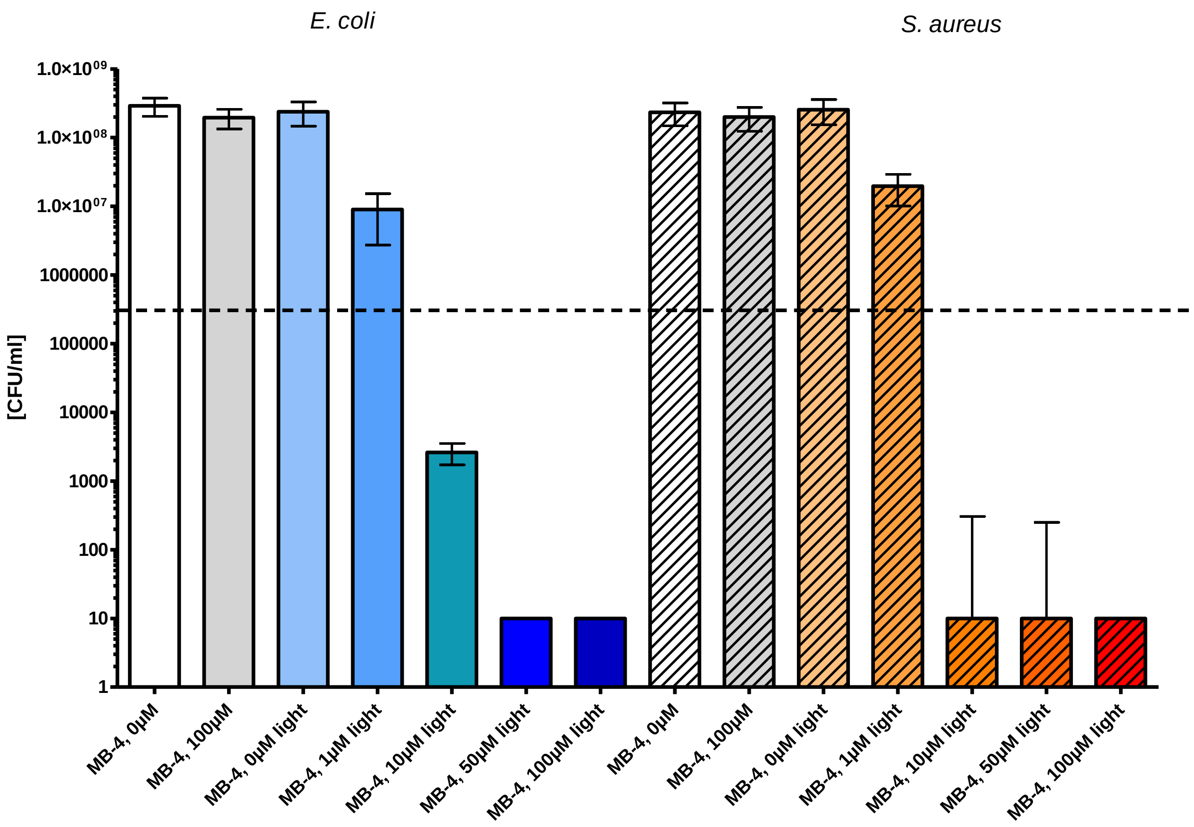

Figure S-17: Photodynamic inactivation of *E. coli* and *S. aureus* by methylene blue derivative MB-4.

Photodynamic treatment was performed using different concentrations of MB-4 with and without illumination (30 J cm^{-2}). Surviving colonies were counted 24 h later. Black dashed line corresponds to a reduction of 3 log_{10} steps in bacteria numbers (99.9% killing efficacy). White and grey column: controls without illumination. Coloured columns: MB-4 + light activation, blue: *E. coli*; crosshatched: *S. aureus*. (n=3 experiments: mean values ± standard deviation)

MB-5

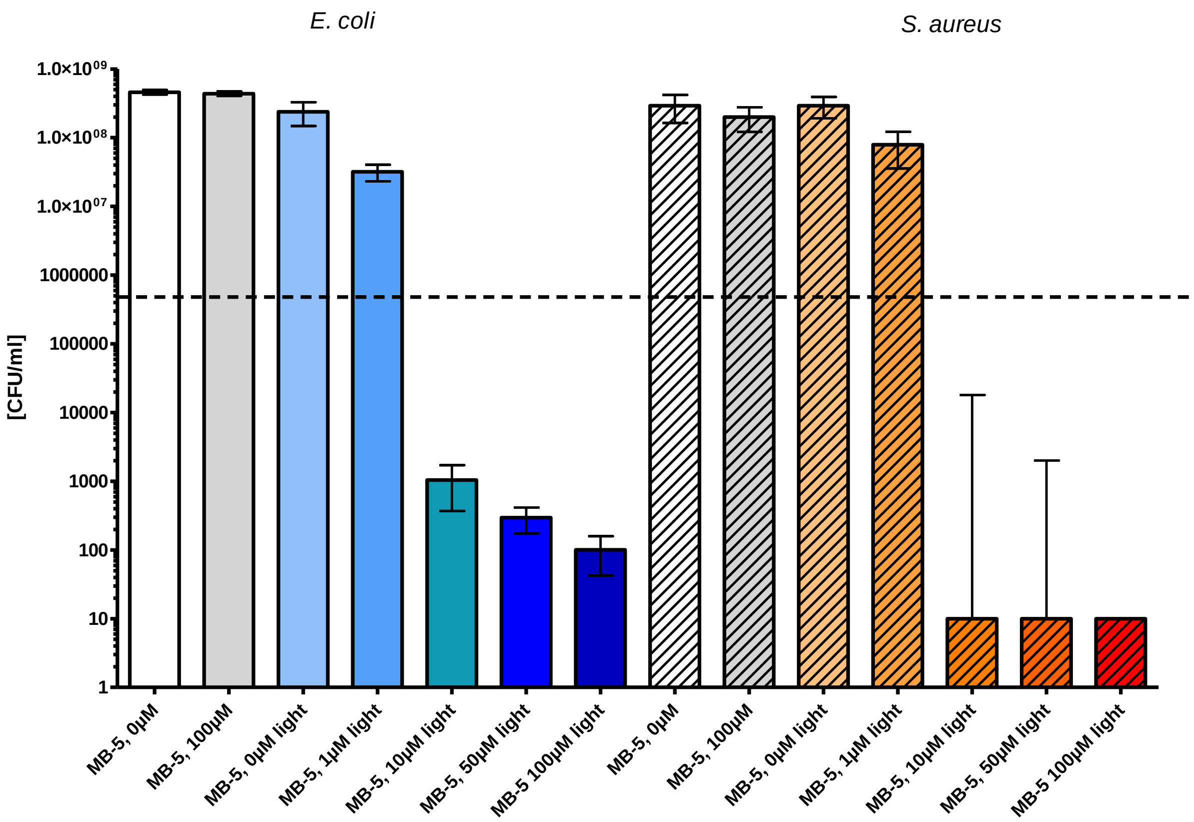

Figure S-18: Photodynamic inactivation of *E. coli* and *S. aureus* by methylene blue derivative MB-5.

Photodynamic treatment was performed using different concentrations of MB-5 with and without illumination (30 Jcm^{-2}). Surviving colonies were counted 24 h later. Black dashed line corresponds to a reduction of 3 $\log_{10}$ steps in bacteria numbers (99.9% killing efficacy). White and grey column: controls without illumination. Coloured columns: MB-5 + light activation, blue: *E. coli*; crosshatched: *S. aureus*. (n=3 experiments: mean values ± standard deviation)

MB-6

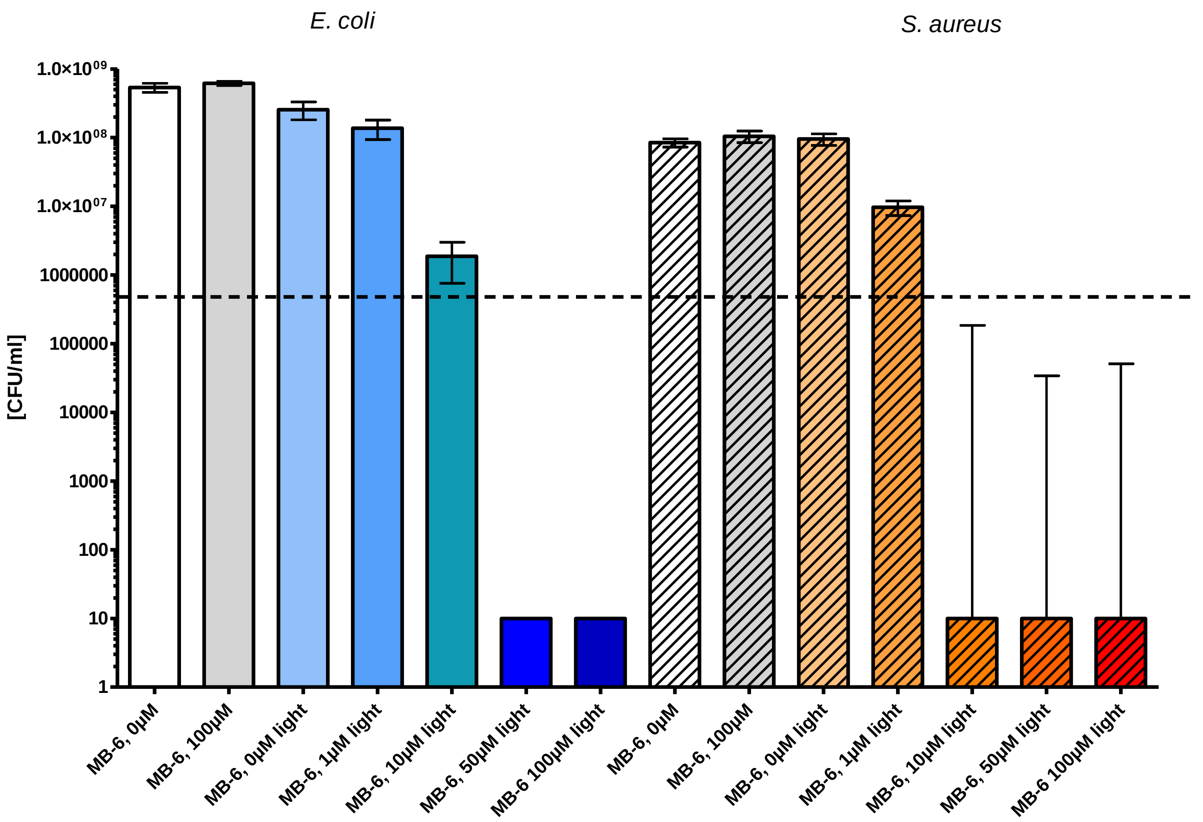

Figure S-19: Photodynamic inactivation of *E. coli* and *S. aureus* by methylene blue derivative MB-6.

Photodynamic treatment was performed using different concentrations of MB-6 with and without illumination (30 J cm^{-2}). Surviving colonies were counted 24 h later. Black dashed line corresponds to a reduction of 3 log_{10} steps in bacteria numbers (99.9% killing efficacy). White and grey column: controls without illumination. Coloured columns: MB-6 + light activation, blue: *E. coli*; crosshatched: *S. aureus*. (n=3 experiments: mean values ± standard deviation)

---

[i] literature known: H. Li, M. Hao, L. Wang, W. Liang, K. Chen, *Org. Prep. Proced. Int.* **2009**, *41(4)*, 301-307.
[ii] for preparation see: K.-Q. Ling, L.M. Sayre, *J. Org. Chem*. **2009**, *74*, 339-350.
[iii] literature known: N. Fomina, C. McFearin, M. Sermsakdi, O. Edigin, A. Almutairi, *J. Am. Chem. Soc.* **2010**, *132*, 9540-9542.
[iv] literature known: B. Wilson, M.J. Fernández, A. Lorente, K.B. Grant, *Org. Biomol. Chem.*, **2008**, *6(21)*, 4026-4035.
[v] For preparation see: L. Strekowski, D-F.Hou and R. L. Wydra, *J. Heterocyclic Chem.*, **1993**, *30*, 1693-1695.
[vi] TFA salt literature known, see: D.S.Crumrine, M.D.Choubal, J.R.Kanofsky, J.J.Feigenbaum, *Neurochem. Res.***1997**, *22*, 107-111; D.S. Crumrine, M.D. Choubal, J.R. Kanofsky, J.J. Feigenbaum, K. Payza, *Peptides* **1996**, *17*, 991-994; O.M. New, D. Dolphin, *Eur. J. Org. Chem.* **2009**, 2675-2686.
[vii] Iodide salt literature known: L. Strekowski, D-F.Hou and R. L. Wydra, *J. Heterocyclic Chem.*, **1993**, *30*, 1693-1695.
